# Tracing the Origin of Korean Invasive Populations of the Spotted Lanternfly, *Lycorma delicatula* (Hemiptera: Fulgoridae)

**DOI:** 10.3390/insects12060539

**Published:** 2021-06-10

**Authors:** Hyojoong Kim, Sohee Kim, Yerim Lee, Heung-Sik Lee, Seong-Jin Lee, Jong-Ho Lee

**Affiliations:** 1 Animal Systematics Laboratory, Department of Biology, Kunsan National University, Gunsan 54150, Korea; veritas72@kunsan.ac.kr; 2 Animal & Plant Quarantine Agency, Gimcheon 39660, Korea; lhsgo@korea.kr (H.-S.L.); mycomania21@korea.kr (S.-J.L.); acarologist@korea.kr (J.-H.L.)

**Keywords:** exotic species, invasion route, inferring origin, molecular epidemiology, population genetics

## Abstract

**Simple Summary:**

*Lycorma delicatula* (White), the spotted lanternfly (SLF) is known to be native to China, India, and Vietnam, but after the first detection in 2004 as invasive in Korea, it was also found in Japan in 2008 and the United States in 2014. As a polyphagous sap-sucking insect, the SLF seriously damages the host plant by sucking phloem sap and producing honeydew, which causes sooty mold disease on leaves and this damage eventually results in an economic loss by reducing the quality and yield of the fruits. After the emergence and spread of SLF in Korea, it has not been confirmed yet where they came from and how they invaded from the source region. To infer the most likely source population for the Korean invasive populations, we investigated the population structure of SLF including its native region (China) and invasive regions (Japan and Korea) using 15 microsatellite loci which were previously developed. Our study set out to solve the correlation between the source and invasive populations of SLF, especially with regards to identifying artificial transfer possibly reoccurring in other invasive regions.

**Abstract:**

*Lycorma delicatula* (White) suddenly arrived in Korea where it rapidly spread out in the central region of Korea and caused serious damage to grape vineyards. To trace the source region of its invasiveness, population genetic structures were compared between the native region, China, and the introduced regions, Korea and Japan. We examined 762 individuals from 38 different population collections using 15 microsatellite loci. Both principal coordinate and structure analyses displayed that the Chinese populations were separated into three subgroups which were located significantly far apart from each other. Among them, the Shanghai population was located closest to most Korean populations. Based on the genetic relationships and structures, it was revealed that the multiple introductions into Korea occurred at least three times. In addition, the Shanghai population was strongly estimated to be a source of initial invasive populations of Korea. In addition, analysis of the approximate Bayesian computation suggested simultaneous spread from two distant locations early in the invasion by artificial transportation of the host plants bearing egg masses. Our population genetics study can provide a precedent case with regards to identifying spreads by anthropogenic outcomes in other invasive regions.

## 1. Introduction

The occurrence of invasive pests is considerably increasing due to a large degree of human-mediated trades around the world [1,2,3]. An outbreak of invasive pests is sometimes a serious threat to human health, ecosystems, and the economy [1,4,5]. Accordingly, to establish their effective control strategy, determining the geographic origin of invasive pests has significant implications in many ways since many efforts are being made to control invasive pests worldwide. In particular, it is critical to determine the biology of invasive pests in native regions, in order to prevent reintroduction and establish a pest control plan [6,7].

*Lycorma delicatula* (White), the spotted lanternfly (SLF), is known to be native to China, India, and Vietnam [8,9]. SLF is distributed nationwide in China and also distributed in Taiwan, Vietnam, and India where the temperature is relatively higher than in northern China [8,9]. After the first detection of SLF as invasive in Korea, it was also found in Japan in 2008 [10] and the United States in 2014 [11]. This species is a polyphagous sap-sucking insect, which feeds on over 100 plants worldwide, including various fruit trees (e.g., grape and stone fruits) and ornamental plants [12]. The SLF seriously damages the host plant by sucking phloem sap and producing honeydew, which causes sooty mold disease on leaves and this damage eventually results in an economic loss by reducing the quality and yield of the fruits [13]. A vineyard in the United States that was recently damaged by the SLF showed a 90% yield loss of grapes due to the falling-off in the quality of fruits [14]. Furthermore, a serious infestation of SLF led to a decline in fruit production and even an effect on plant death [14]. 

The SLF is a recent invasive pest in Korea [15]. After its first detection in 2004 [15], it rapidly spread mainly in the central region of Korea [16,17]. Until 2009, this species was found mostly in Seoul and metropolitan areas [15,16]. However, this species is currently distributed everywhere in mainland Korea [18]. Subsequently, SLF is causing great damages to agriculture as well as wild forests [10,19]. In particular, continuing outbreaks brought large damages to cultivated grapes. In the initial five years of the invasion, SLF damaged about one ha of vineyards, but in 2010, the damage increased to 8478 ha [18]. The successful settlement of this species is thought to be due to the success of overwintering and is considered to be related to the recent increase in winter temperatures in Korea [17]. The high number of hosts available to SLF in Korea was likely one of the factors that let SLF rapidly spread out. According to the previous study, this species utilizes 41 host plants, including 38 woody and three herbaceous species [16]. In particular, the main winter host of SLF, the native Chinese tree, called the ‘tree of heaven’, *Ailanthus altissima*, is widely distributed in Korea along roadsides, which allows SLF to proliferate everywhere [17,20]. 

After the emergence and spread of SLF in Korea, it has not been confirmed yet where they came from and how they invaded from source region to invasive region. The invasion source of the Korean SLF population has been suggested to be China [10,15,19], but this has not been thoroughly verified. Although SLF has a wide distribution in China [8,9] and is geographically close to Korea, it is unlikely that it would have arrived across the sea since its flight ability is very poor [21,22]. The typical mode of dispersal of SLF is a short distance plant to plant flights, averaging 20 m at a time [21]. Such a dispersal pattern seems improper to fly a long-distance migration route such as across the sea. In Japan, this species has been recorded in Okinawa, Honshu, and Kyushu since 1930, and it was only sporadically observed [23]. However, the SLF suddenly appeared in the middle part of mainland of Japan in 2008 [10]. This sudden occurrence supports the assumption that the SLF was artificially transferred by exported products, probably as egg mass and not by natural migration.

Under this assumption, a population genetic study to trace the origin of SLF was conducted by Kim et al. [10], revealing that Korean individuals are identical to Chinese individuals collected from Beijing, Tianjin, Qingdao, and Shanghai based on the two mitochondrial DNA regions, NADH dehydrogenase subunit 2 and NADH dehydrogenase subunit 6. Yet, there was a limit to the discussion of the invasion route of SLF since this study only included 18 specimens of SLF and the two mtDNA markers have low resolution [10]. More recently, two population genetic studies using microsatellite showed good power of discrimination on the genetic relationships between the local populations [24,25]. Park et al. [25] showed that there are at least three genetically distinct populations in Korea, possibly meaning multiple introductions of SLF. Unfortunately, further discussion of the origin was not possible in this study, since there was no comparison with a source population. In the results of population genetics in China [24], the native region of SLF, four genetically different populations were identified. Interestingly, this study revealed that in SLF, migration predominantly occurred by some populations rather than all populations [24]. However, it is not known how these characteristics relate to the SLF invading the regions other than the place of origin. 

In this study, we addressed some questions concerning the invasion of SLF in East Asia by focusing on identifying the geographic origin and invasive route of SLF, especially weighting on the possibility of introduction from China: (1) Did the SLF come from China? (2) If SLF came from China, where did it specifically come from? (3) Has the SLF been introduced in Korea several times independently? (4) Where did the central Japanese group come from? (5) Is there any connection with the Korean group? Therefore, we investigated the population structure of SLF, including its native region (China) and invasive regions (Japan and Korea), using 15 microsatellite loci which were previously developed [26]. We characterized the genetic diversity of SLF populations from the three countries and compared the genetic differentiation among the regional populations. We also inferred the most likely source population for the Korean invasive populations by using Bayesian inference methods such as approximate Bayesian computations (ABC), which can estimate the relative likelihood of alternative introduction of the SLF with comparison of complex scenarios encompassing some introduction processes. Our study seeks to solve the correlation between the source and invasive populations of SLF, especially with regards to identifying artificial transfer possibly reoccurring in other invasive regions.

## 2. Materials and Methods

### 2.1. SLF Samples

SLF samples of the native area were collected from 12 localities in China (Table 1). SLF samples of the invasive area were collected from 25 localities in Korea and one locality in Japan (Table 1). We performed dense specimen collection throughout mainland Korea because SLF is currently distributed in almost all regions in Korea. All insect samplings have been carried out in unrestricted areas where collection permits are not required. In total, we examined 762 individuals obtained from 38 populations. Adult and nymph samples were collected using an insect net. To avoid the chance of sampling individuals from the same hatching, each individual of SLF was collected from single host plants (*Altissima ailanthus*) more than 3 m away from one another. All the fresh SLF samples used for molecular analyses were preserved in 95% or 99% ethanol and stored at −70 °C.

### 2.2. Microsatellite Genotyping

A total of 762 individuals were genotyped using 15 microsatellite loci (Lde01 to Lde15) which were previously isolated from SLF [26]. In a preliminary test, all loci developed in the previous study [26] were polymorphic among most population samples and were included in the henceforth analyses.

Total genomic DNA was extracted from single individuals using LaboPass™ Tissue Genomic DNA mini Kit (COSMOGENETECH, Seoul, Korea) according to the manual protocol. To avoid the contamination of exotic genomic DNA (bacteria, parasites, endosymbionts, etc.), we only used muscle tissues extracted from ptero-thoracic parts. The muscle tissues were left in the lysis buffer with protease K solution at 55 °C for 24 h and the cleared cuticle was dehydrated. Microsatellite amplifications were performed using AccuPower^®^ PCR PreMix K-2037 (BIONEER, Daejeon, Korea) in 20 µL reaction mixtures containing 0.5 µM forward labeled with a fluorescent dye (6-FAM, HEX, or NED), reverse primers and 0.05 µg of DNA template. PCR was performed using a GS482 thermo-cycler (Gene Technologies, Essex, UK) according to the following procedure: initial denaturation at 95 °C for 5 m, followed by 34 cycles of 95 °C for 30 s; annealing at 56 °C for 40 s; extension at 72 °C for 45 s, and a final extension at 72 °C for 5 m. PCR products were visualized by electrophoresis on a 1.5% agarose gel with a low range DNA ladder to check for positive amplifications. Automated fluorescent fragment analyses were performed on the ABI PRISM 377 Genetic Analyzer (Applied Biosystems, Waltham, MA, USA), and allele sizes of PCR products were calibrated using the molecular size marker, ROX labeled-size standard (GenScan^TM^ ROX 500, Applied Biosystems, Waltham, MA, USA). Raw data on each fluorescent DNA products were analyzed using GeneMapper^®^ version 4.0 (Applied Biosystems, Waltham, MA, USA).

### 2.3. Data Analysis

Allele data analysis results were processed in GENALEX 6.503 [27] through Microsoft Office Excel 2013 (Microsoft). Observed (*H*_O_) and expected heterozygosity (*H*_E_) values among loci were estimated using GENEPOP 4.0.7 [28] among the population datasets as well as between the USA and Asian datasets. Levels of significance for Hardy–Weinberg equilibrium (HWE) and linkage disequilibrium tests were adjusted using sequential Bonferroni correction for all tests involving multiple comparisons [29]. Deviations from HWE were tested for heterozygote deficiency or excess. MICRO-CHECKER [30] was used to test for null alleles [31] and identify possible scoring errors because of the large-allele dropout and stuttering. The program FSTAT 2.93 was used to estimate the gene diversity (*H*_S_), a mean number of alleles (*N*_A_), and allelic richness (*R*_S_). 

Groupings based on some biogeographical (spatial) and temporal groups were tested independently with analysis of molecular variance (AMOVA) [32] in ARLEQUIN 3.5.1.2 [33], with significance determined using the non-parametric permutation approach described by Excoffier et al. [32]. We also used ARLEQUIN for calculations of pairwise genetic differentiation (*F*_ST_) values [34], in which 38 populations were assigned by each local collection. Exact test of population differentiation was conducted as optioned by 100,000 Markov chains, 10,000 Dememorization steps, and 0.05 significance level.

To examine genetic relationships between populations of SLF, we used Principal Coordinate Analysis (PCoA) on a genetic distance matrix based on codominant genotypic distance [35,36] provided in GENALEX 6.503 [27]. The PCoA is a multivariate technique that allows us to find and plot the major patterns within a multivariate dataset (e.g., multiple loci and multiple samples) [35,36].

The program BOTTLENECK 1.2.02 [37] was used in the attempt to recognize in our samples the effect of a recent bottleneck, separately for each population. Two mutation models, considered appropriate for microsatellites [38], were applied as the strictly stepwise mutational model (SMM) and the two-phase model (TPM). For the TPM, a model that includes both 90% SMM and 10% TPM was used for 20,000 iterations. Significant deviations in observed heterozygosity over all loci were tested using a nonparametric Wilcoxon signed-rank test [37].

We performed two analyses to confirm dispersal and Isolation-By-Distance (IBD) using two different datasets of the Korean and Chinese SLF. The Korean dataset included 17 regional populations collected only in 2010 and 2011 while the Chinese dataset consists of all 12 regional populations (Table 1). First, the level of the nation-scale spatial autocorrelation [36,39,40] was analyzed. An individual-by-individual genetic distance matrix [35,36] was generated along with a subsequent geographic distance matrix. The autocorrelation coefficient, *r*, is calculated through a multivariate approach (i.e., across all loci) and equals the genetic similarity between two individuals. The statistical significance of the spatial autocorrelation hypothesis is determined by generating 95% confidence intervals from 10,000 random permutations. Values of *r* that lie within the confidence intervals fail to reject the null hypothesis of no spatial genetic structure. Restricted dispersal is suggested when *r* > 0; alternatively, when *r* < 0, large-scale dispersal is implicated [40]. For further evaluation, support for the estimation of *r* is given through the bootstrapping procedure. According to the geographic scale, we performed analyses at different distance size classes, ‘10 and 20 km’ in the Korean dataset and ‘20 and 40 km’ in the Chinese dataset, with 999 permutations and bootstrapping of *r* with 1000 times. Second, the significance of the regression of codominant genotypic distance (GD) on geographic distance (GGD) was tested using the Mantel procedure [41], permuting 1000 times. Spatial autocorrelation and Mantel tests were run using the program GENALEX v6.503 [27]. In addition, the population relative (*r*) test, with 99 permutations for 100 bootstraps, was performed to confirm the correlation between GD and GGD for 30 regional populations from Korea (17), China (12), and Japan (1) in GENALEX.

STRUCTURE 2.3.4 was used to analyze the genetic structure of 38 SLF populations using a Bayesian clustering approach [42]. We set the number of clusters (*K*) from 1 to 11 and conducted 5 independent runs for each value of *K*. Each run consisted of a burn-in period of 30,000 steps, followed by 500,000 Markov chain Monte Carlo (MCMC) repetitions with a model allowing admixture. The Δ*K* value calculated as Δ*K* = m(|*L*″(*K*)|)/s[*L*(*K*)] was obtained using the ad hoc quantity, which is calculated based on the second-order rate of change of the likelihood [43]. To correctly perform this process, ∆*K* was calculated using the online resource STRUCTURE HARVESTER 0.6.94 [44] that explained the structure in data. Visualization of the STRUCTURE results was conducted using DISTRUCT 1.1 [45]. In addition, GENECLASS 2 was used to perform the assignment/exclusion tests, which were used for the detection of genetic signatures of dispersal and immigration [46]. For each individual of a population, the program estimated the probability of belonging to any other reference population or to be a resident of the population where it was sampled. The sample with the highest probability of assignment was considered as the most likely source for the assigned genotype. We used a Bayesian method of estimating pop. allele frequencies [47] with Monte Carlo resampling computation (10,000 simulated individuals) in order to infer the significance of assignments (type I error, alpha = 0.01) [48].

To estimate the relative likelihood of alternative introduction scenarios of the SLF, an approximate Bayesian computation (ABC) was performed for microsatellite data as implemented in DIYABC 1.0.4 [49]. DIYABC allows the comparison of complex scenarios involving bottlenecks, serial or independent introductions, and genetic admixture events in introduced populations [6]. The parameters for modeling scenarios are the times of split or admixture events, the stable effective population size, the effective number of founders in introduced populations, the duration of the bottleneck during colonization, and the rate of admixture [50]. The software generates a simulated dataset used to estimate the posterior distribution of parameters in order to select the most likelihood scenario [50]. DIYABC generates a simulated dataset that is then used to select those most similar to the observed dataset and the so-called selected dataset (*n_δ_*), which is finally used to estimate the posterior distribution of parameters [49]. The DIYABC 1.0.4 analysis was conducted for the purpose of testing the initial introduction process of SLF from the source to invasive regions. Since multiple introductions were delivered based on the results of PCoA, STRUCTURE, and GENECLASS2 (see Results), several populations could be limitedly estimated as the source of the invasive relationships. Therefore, the ABC analyses were used to confirm the various scenarios for simulating the introduction route between the source (China) and invasive populations (Korea), which were selected from a large number of population collections in 2010. We set one source group (SG), Shanghai (CN10-SH), and two invasive groups, a northern invasive group (NG) of Korea (KR10-SL, KR10-IN, KR10-SW) and a middle group (MG) of Korea (KR10-CA, KR10-NS, KR10-BA). Four scenarios (1–4) were estimated with comparison to each other in the DIYABC (Supplementary Information Figure S1). We produced 1,000,000 simulated datasets for each scenario. We used a generalized stepwise model (GSM) as the mutational model for microsatellites, which assumes increases or reductions by single repeat units [49]. To identify the posterior probability of these three scenarios, the *n_δ_* = 40,000 (1%) simulated datasets closest to the pseudo-observed dataset were selected for the logistic regression, which are similar to the *n_δ_* = 400 (0.01%) ones for the direct approach [50]. The summary of statistics was calculated from the simulated and observed data for each of the tested scenarios, including the mean number of alleles per locus (*A*), mean genetic diversity for each group and between groups, genetic differentiation between pairwise groups (*F_ST_*), classification index, shared alleles distance (*D_AS_*), and Goldstein distance.

## 3. Results

### 3.1. Genetic Diversity within Populations

We successfully genotyped 762 SLF individuals using 15 microsatellite loci, which were found as all non-clonal MLGs (Table 2). Observed heterozygosity (*H*_o_) and expected heterozygosity (*H*_E_) values in Korean populations ranged from 0.172 to 0.500 and from 0.171 to 0.513, respectively, whereas in Chinese populations, *H*_o_ and *H*_E_ values ranged from 0.413 to 0.548 and from 0.423 to 0.608, respectively. There was no evidence of significant linkage disequilibrium or frequency of null alleles. In HWE, there were significant deviations in KR09-GW, CN10-LA by heterozygote deficit, which was likely due to retaining numerous unique genotypes with private alleles within a population related to their relatively high *H*_E_. Values related to genetic (allelic) diversity were shown as generally higher throughout the native region than in the invasive regions. Gene diversity (*H*_S_) and mean number of alleles (*N*_A_) in Korean populations averaged 0.45 and 3.69, respectively, which were lower than those of Chinese populations, 0.51 and 4.26, respectively. Similarly, allelic richness (*R*_S_, mean ± s.d., 2.63 ± 0.31) in Korean populations was slightly lower than *R*_S_ (2.87 ± 0.38) in Chinese populations. Inbreeding coefficients (*F_IS_*) showed a non-significant difference between observed and expected heterozygosity in most populations, but some initial populations such as KR07-SL, KR09-GB, KR09-GW, etc. had relatively high *F_IS_* values in the invasive area.

### 3.2. Genetic Variation, Differentiation between Populations, and Bottleneck

To confirm genetic variance between the preordained groups, four cases were analyzed using AMOVA implemented in ARLEQUIN [33] (Table 3). Genetic variance among groups (Va) in case 1 (grouped by year) was the lowest among all the cases at about 0.48%; it was similarly low at about 4–6% in case 2 (grouped by country) and case 3 (grouped by year and country). However, genetic variance among groups at about 10% in case 4 (grouped by genetic structure (*K* = 4)) suggests that there are relatively different regional structures in combination of variation over time.

We estimated pairwise genetic differentiation (*F*_ST_) among 38 different geographical populations (Table S1). Pairwise comparisons of *F*_ST_ values showed that Korean populations were genetically closer to each other than Chinese populations. Mean *F*_ST_ within Korean populations was 0.069, whereas mean *F*_ST_ within Chinese populations was 0.157. *F*_ST_ between Korean and Chinese populations averaged 0.143, while *F*_ST_ between Korean and Japanese populations and between Chinese and Japanese populations averaged 0.129 and 0.197, respectively. Surprisingly, *F*_ST_ values between CN10-SH and 13 Korean populations (KR09-JE, KR09-GB, KR09-GW, KR10-SL, KR10-IN, KR10-NY, KR10-SW, KR10-IC, KR10-AS, KR10-CA, KR10-CY, KR10-NS, KR10-GC, KR10-GJ, KR10-BA, and KR10-KJ) were relatively low (averaging 0.012), which was lower than *F*_ST_ within Korean populations (0.069).

**Figure 1:**
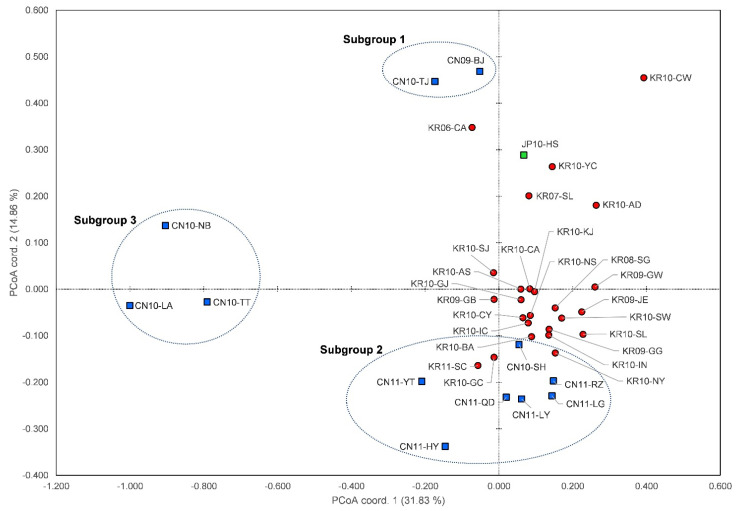
A plot of the principal coordinate analysis based on the first two factors for 38 populations of SLF in GENALEX. Each color corresponds to the countries: red—Korea with 25 populations; blue—China with 12 populations; green—Japan with 1 population.

A result of PCoA (Figure 1) estimated by codominant-genotypic distance showed that the plots of the Korean populations were concentrated in the first quadrant, but those of the Chinese populations were separated into three subgroups and located apart from each other. Interestingly, among the Chinese populations, CN10-SH was located between most Korean population plots. Within the Chinese populations, CN10-NB, CN10-LA, and CN10-TT, which were close to each other, were found to be genetically different from most other regional groups. The plot of JP10-HS was located close to those of several populations in Korea.

Although the bottleneck test should be cautiously regarded because the sample size for some populations was less than 30 individuals [37], significant signatures of genetic bottlenecks using the program BOTTLENECK were tested in this study, especially for estimation of Korean and Japanese populations (Table S2). Using either the SMM and TPM, evidence of a recent population bottleneck (*p* < 0.05 for Wilcoxon signed-rank tests) was detected in 19 invasive populations (KR08-SG, KR09-GG, KR09-JE, KR09-GW, KR10-SL, KR10-IN, KR10-NY, KR10-SW, KR10-IC, KR10-CA, KR10-CY, KR10-NS, KR10-GC, KR10-BA, KR10-KJ, KR11-SC, CN10-SH, CN10-NB, and CN10-LA). In addition, the mode shift revealed a recent bottleneck in six invasive populations (KR07-SL, KR09-GB, KR10-KJ, KR10-SJ, KR10-CW, and JP10-HS). Only KR10-KJ was estimated to bottleneck in both the SMM and mode shift.

### 3.3. Spatial Autocorrelation and Isolation-by-Distance

In order to confirm the spatial distribution of each of the SLF groups in China and Korea, spatial autocorrelation was tested through spatial structure analysis by GENALEX 6.503 [27]. The graph of a spatial autocorrelation (Figures S1–S4) means that the *r* (correlation coefficient) of the Y-axis indicates that the genetically related group inferred to be distributed in a geographic boundary as the first initial section with a positive value. First, for the Korean group, the Mantel test for IBD to examine the correlation between genetic and geographic distance of the samples showed significantly lower correlation, with *r* = 0.078 (*p* = 0.010), than for the Chinese group (see below). When GD and GGD were pairwise compared in each sample at 10 km class (Figure S1) and 20 km class (Figure S2), significantly positive spatial autocorrelation at distances up to ca. 80 km, with the highest correlation coefficient within the first 10 km and 20 km, respectively, was detected even though the bootstrapping of *r* values slightly overlapped with the 95% confidence interval of the null hypothesis at 10 km class. Consequently, the distribution of the SLF in Korea had IBD correlation within a spatial distribution of ca. 80 km. This indicates that the SLF is formed of genetically related groups that show the IBD-correlated patterns within the range of about 80 km, where both geographic and genetic differences among Korean populations are correlated. Second, for investigating the correlation between genetic and geographic distances among the Chinese samples, the Mantel test for IBD showed a positive correlation, with *r* = 0.426 (*p* = 0.010). When GD and GGD were pairwise compared in each sample at 20 km class (Figure S3) and 40 km class (Figure S4), significantly positive spatial autocorrelation at distances up to ca. 260 km in both classes, with the highest correlation coefficient within the first or second 40 km, respectively, were detected. Consequently, the distribution of the SLF in China was within a spatial distribution of at least ca. 260 km, where the regional populations have been shown to be correlated with IBD. This indicates that the SLF population is formed of genetically related groups that show genetically homogeneous patterns (similar genetic structure) in the geographic range of approximately a province-scale in China, where both geographic and genetic differences among Chinese populations are correlated. This analysis showed that if each SLF group is collected in a range exceeding ca. 260 km, it would be genetically different from the other groups by the geographic isolation. Therefore, this result corroborated the genetic differences between the three SLF groups from the three regions, Beijing and Tenjin (BJ + TJ), Shandong (SD), and Zhejiang (ZJ). In addition, the population relative test was tested to confirm the correlation of IBD in each population using GENALEX 6.503. 

**Figure 2:**
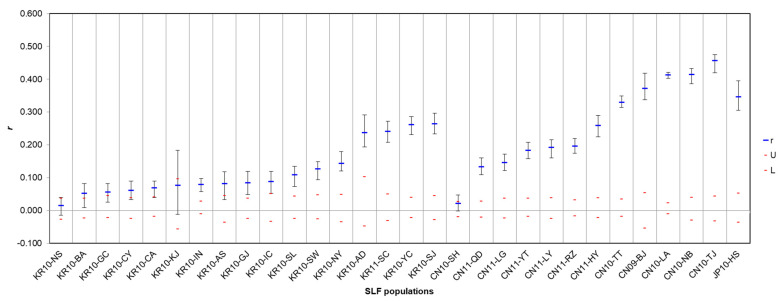
Population relative (*r*) test for 30 regional populations from Korea (17), China (12), and Japan (1) in GENALEX. r (blue) means autocorrelation coefficient; U (upper red) is +95% confidence; L (lower red) is −95% confidence; cross bars are standard deviation.

The population relative test can be used to show the ideal IBD correlation when the genetic distances of one group vs. all other groups are positively correlated with each other; that is, when the *r* value is significantly large (Figure 2). In contrast, there is a high probability that it was either a source or invasive site (Figure 2) if the *r* value is very small when the correlation compared between the origin and invasion sites. Therefore, CN10-SH with the smallest *r* value in the origin area is highly likely to be the source population in China, and KR10-NS with the smallest *r* value in the invasive area is highly likely to be the initial invasive population in Korea (Figure 2), which is a similar concept in application of the correlation between GD and GGD for tracing the origin of invasive pest previously studied by Lozier et al. [51].

### 3.4. Genetic Structure and Assignment

**Figure 3:**
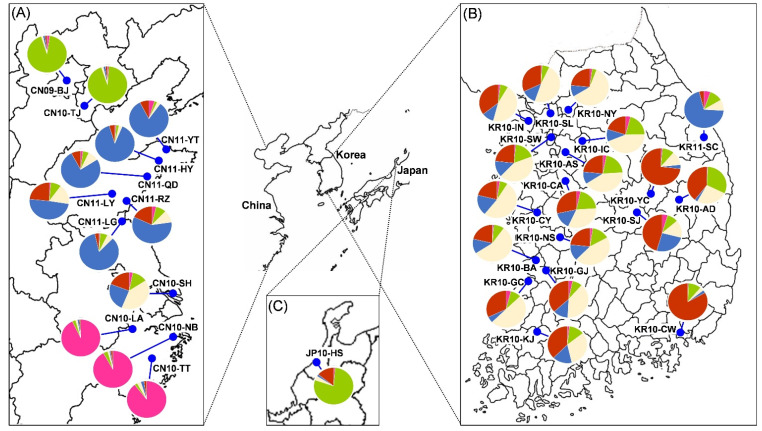
Sampling locations for 31 populations of SLF in (**A**) China (12 populations), (**B**) Korea (18 populations), and (**C**) Japan (1 population). The pie graphs indicate the genetic structure of each local population based on a Bayesian inference of multilocus microsatellite genotype in STRUCTURE, which is partitioned when *K* = 5.

In the STRUCTURE analyses, the most likely number of clusters was *K* = 2 using the Δ*K* calculation from *K* = 1 to *K* = 11 [43]. However, Evanno, Regnaut, and Goudet [43] previously emphasized that while Δ*K* was useful in identifying the correct number of clusters, it should be used together with the battery of other information provided by STRUCTURE. Thus, we decided that the results of STRUCTURE analyses were demonstrated when *K* = 2, 3, 4, 5, considering both L(K) and Δ*K*. In the all STRUCTURE results (*K* = 2, 3, 4, 5; Figures S5 and S6), CN10-NB + CN10-TT + CN10-LA had a dominant ‘pink’ assignment. The genetic structure when *K* = 3, 4 showed that most of the Korean populations and CN10-SH indicated similar pattern of assignments having more dominant ‘white’, while KR11-SC and the Chinese populations collected in 2011 showed a mixture but more dominant ‘blue’. The genetic structure when *K* = 5 (Figure 3) displayed that CN09-BJ, CN10-TJ, and JP10-HS had a similar dominant ‘green’ assignment while KR10-AD and KR10-CW had a dominant ‘red’ assignment.

The Bayesian assignment tests using GENECLASS 2 were carried out to identify population membership of individuals from all populations (Table S3). The result of the assignment test indicated the average probability with which individuals were assigned to the corresponding reference population. The self-assignment probability values averaged 0.380 ± 0.07 (mean ± s.d.) in Korean populations, and 0.525 ± 0.03 in Chinese populations. This assignment approach also could be used to interpret the inference of invasion of SLF genotypes into Korea from China. From Chinese to Korean populations, assignment probability values averaged 0.116 ± 0.118. Similar to the results of STRUCTURE (Figure 3), among Chinese populations, CN10-SH had the highest assignment probability values, averaging 0.337 ± 0.112 from most Korean populations.

### 3.5. Inferring an Invasive Route by Testing Hypothetical Scenarios by ABC Analysis

**Figure 4:**
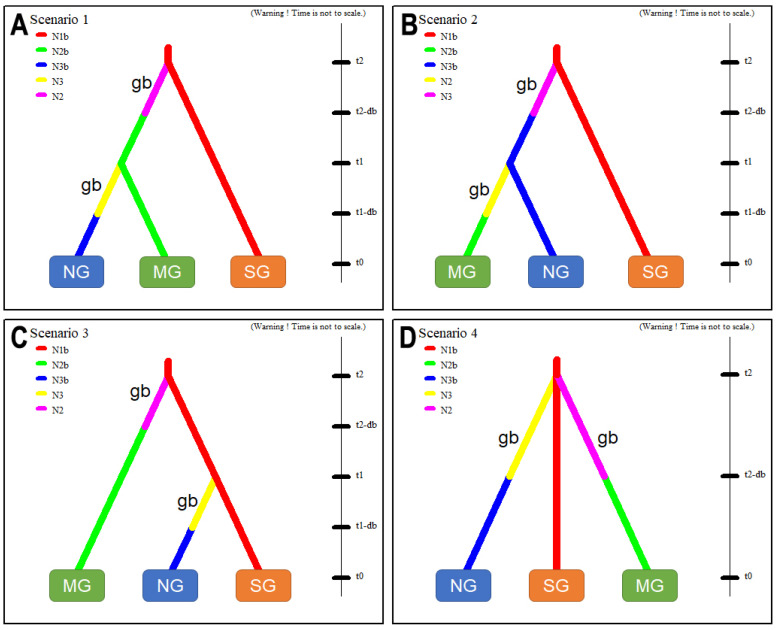
The four scenarios (**A**–**D**) for the DIYABC analyses to infer the invasion route into northern (NG) and/or middle (MG) part of Korea from Shanghai (SG), China, using a dataset that includes 168 individuals from three subgroups, which consisted of 80 individuals from the ‘NG’ group (KR10-SL, KR10-IN, KR10-SW); 62 from the ‘MG’ group (KR10-CA, KR10-NS, KR10-BA); and 26 from the ‘SG’ group (CN10-SH). ‘gb’ means genetic bottleneck.

To propose the most likely scenario of an invasive route of the SLF when introduced to Korea, ABC tests were conducted with four scenarios (Figure 4). The dataset included three different groups as one source group (SG) and two invasive groups (NG, MG) (see Materials and Methods). These results were presented as a logistic regression using DIYABC software, estimating the posterior probability of each tested evolutionary scenarios of invasion for the selected simulated data (*n_δ_*) [49], which ranged between 4000 and 40,000 *n_δ_*. 

As derived from the results (Figure S7), scenario 1 (Figure 4A) obtained the highest posterior probability, ranging from 0.021 (*n_δ_* = 4000) to 0.036 (*n_δ_* = 40,000) with a 95% CI of 0.006–0.036 and 0.028–0.043, which assumes NG recently diverged from MG and both previously diverged from SG. Scenario 2 (Figure 4B), which assumes MG recently diverged from NG and both previously diverged from SG, showed a posterior probability ranging from 0.019 (*n_δ_* = 4000) to 0.035 (*n_δ_* = 40,000) with a 95% CI of 0.006–0.032 and 0.028–0.043. Scenario 3 (Figure 4C), which assumes MG recently diverged from SG and NG previously diverged from SG, showed a posterior probability ranging from 0.028 (*n_δ_* = 4000) to 0.047 (*n_δ_* = 40,000) with a 95% CI of 0.010–0.047 and 0.024–0.036. Scenario 4 (Figure 4D), which assumes MG and NG simultaneously diverged from SG when introducing, showed a posterior probability ranging from 0.932 (*n_δ_* = 4000) to 0.900 (*n_δ_* = 40,000) with a 95% CI of 0.904–0.961 and 0.886–0.914.

The ABC simulations based on the four alternative scenarios (1–4) for tracing the invasion route of the SLF gave strong support to the scenario 4 (mean posterior probability: 0.900–0.932) while the remaining other three scenarios (1–3) showed much lower posterior probabilities (<0.036; Figure S7). These suggest that scenario 4 appeared as the most robust hypothesis, presenting the highest posterior probability among the four scenarios tested (Figure 4).

## 4. Discussion

### 4.1. Genetic Structures of SLF in the Native and Invasive Areas

Zhang et al. [24] found that the Chinese SLF population is divided into four subgroups, including three subgroups north of the Yangtze River and one subgroup south of the Yangtze River. Our results provide a well-resolved genetic structure of SLF in its native and invasive regions. We confirmed that there are three subgroups, ‘BJ + TJ’ (Subgroup 1), ‘SD + JS + SH’ (Subgroup 2), and ‘ZJ’ (Subgroup3), in the native population (Figure 1 and Figure 3). Our results are consistent with the previous finding that SLF shows genetic differences to the north and south of the Yangtze River rather than geographic distance [24]. Subgroup 2 (SD + JS + SH) and subgroup 3 (ZJ) are geographically separated from each other by the Yangtze River, which seems to be the restriction of gene flow. In addition, significant genetic differences among the subgroups based on geographic isolation are very consistent, with the former results showing significant morphological differences in wings in the morphometric analysis [19]. Similar to the previous studies [10,24], the SLF populations were divided into several subgroups by the Yangtze River and east–west boundaries in China. However, since our sampling did not cover the western China subgroup (i.e., Shaanxi and Gansu) [24], only three subgroups were identified (Figure 1 and Figure 3).

The Korean and Japanese populations showed relatively low genetic diversity, a typical low genetic diversity in the invasive populations [51,52,53]. Accordingly, genetic bottlenecks were rarely found in the native region, but most of the populations in Korea were found to have genetic drift due to the founder effect of the early invasive population (Table S2). In the Korean SLF population, it could be confirmed that there were traces of simple genotype distribution of the SLF population over a wide area to spread and the impact of outbreaks caused by anthropogenic factors such as transportation in the short period following the invasion process. The genotype of most of the samples spread predominantly in Korea was similar to that of CN10-SH sampled in Shanghai, China. In Korea, compared to the large area in which SLF was distributed, the genetic diversity was relatively low and there was no significant genetic difference according to geographic distance or based on IBD correlation. In contrast, JP10-HS found initially in Japan was genetically close to CN09-BJ and CN10-TJ in China (Figure 3; Table S1). These results are evidence to support the spread of SLF in East Asia by artificial routes from remote regions rather than natural migrations between geographically close regions.

Based on the results of our population genetics (Figure 1 and Figure 3, Figures S5 and S6), most of the populations that invaded and spread to Korea were found to be genetically very similar to subgroup 2 (SD + JS + SH) in China. In Korea, the genetic structure of the samples collected in 2006–2007 was more or less different from the dominant structure of those collected in 2010–2011. The populations collected from 2008 to 2011 in Korea were very similar to CN10-SH except for KR11-SC which, however, was closer to the Shandong group (e.g., CN11-YT, CN11-HY). Overall, most of the SLF populations in Korea appear to have introduced and spread from Shanghai (CN10-SH) while certain populations have independently and intermittently settled in several regions (e.g., KR11-CW, KR11-SC). Similar to our results, the populations sampled in the previous population genetic study of SLF in Korea did not show significant genetic differences between regions, except Samcheok [25]. On the other hand, in the early invasion group, traces of variation due to inland genetic drift were found in the populations such as KR10-AD, KR10-YC, and KR10-SJ. However, in most other regions, the single genetic group close to CN10-SH with a high initial density spread during the multiple introduction process (at least three times) was found to be a cause of forming a relatively simple and dominant genetic structure throughout Korea.

### 4.2. Spatial Distribution of SLF between Origin and Invasion Areas

Based on the results of the spatial autocorrelation (Figures S1–S4) and IBD, SLF is considered a species with very limited migration and spread in natural ecosystems. In China, the geographical structure of spatial distribution of SLF indicated that the genetically related groups were formed within about 260 km of the source area (Figures S3 and S4). As can be seen from the isolation between the Yangtze River, there were topographical elements that could lead the restriction of gene flow under natural conditions (mountain range, river, etc.), affirming that SLF is not a voracious migratory insect [21]. In the early days of the invasion, it was argued that the SLF was introduced by airflow from China to Korea by flight, but it was estimated that flight invasion would be impossible due to SLF’s poor flight capability [21]. The three subgroups of China shown in this study show that gene flow is limited by spatial distribution and that each subgroup is significantly differentiated by geographic isolation. On the other hand, Korea, which is an invasion area, is a smaller space than China and is not far from the first invasion spot(s) to the spread area. The prevalence of SLF groups in 2010 and 2011, which are presumed to have simultaneously invaded and spread from Shanghai to northern and central Korea by ABC analysis estimates, spread in a relatively smaller spatial distribution than the origin, and it was found to be not IBD-correlated by the spatial autocorrelation results (Figures S1 and S2). 

For the SLF to settle and re-spread, the host plant is most essentially limited to the overwintering host, especially *Ailanthus altissima*, so it seems likely that the settling and spread pattern will repeat advantageously around the zone where the host plants were densely cultivated [20,54]. Tree of heaven, *A. altissima*, is commonly used for ornamental landscape trees, street trees, and embankments in China and Korea, so it is pointed out that its high density in the invasive range has been a major cause of the spread of SLF [13]. Interestingly, this species tends to show host preference for various plants for feeding during the nymph period after emerging, as a generalist, but an overwintering host plant such as *A. alitissima* is essential for lay eggs after mating [20]. As the ecological feature of SLF is overwintering through egg masses on the surface of host plants or even artifacts, the artificial spread is possible through vehicle transport, as in the case of the spread of boll weevils in the United States [55]. As with the recent prediction model study [18], the spatial distribution and spread of SLF in Korea are closely related to anthropogenic factors (i.e., human footprint). The prevalence of the initial population seen in the results of the MAXENT analyses [18] was relatively high by the human footprint including transporting activity although it was not a naturally environmental variable. Because of these artificial factors, the rapid spread and spatial distribution of SLF would have been possible despite a short period of invasion, so it is supported that IBD correlation was not significant in Korea in our results.

### 4.3. Introduction Process of SLF from China

Our results suggest that some populations of SLF in around 2009 have been introduced independently and intermittently into Korea, and afterwards, certain populations that appeared to dominate the genetic structure in 2010 had a large spread around that time. At the beginning of the invasion, the insignificant genetic differences and the significance of the bottleneck in the Korean populations support the rapid spread of a certain source population with high initial density. Most of the SLF populations dominantly found in Korea were due to the spread of individuals native to Shanghai, but apart from these, genetically similar individuals to Beijing and Tianjin were in Cheonan in 2006 (similar to the 2010 Japanese population), and those matching the Shandong region appear to have been found in Samcheok in 2011. Interestingly, considering that similar genotypes were occurring nationwide in Korea in 2010 and 2011, it is not possible to rule out continuous introductions from China, especially Shanghai, to Korea by the bridgehead effect [56] rather than the re-proliferation of populations that initially entered Korea. In conclusion, it was estimated that the various SLF populations introduced to Korea had at least three independent introductions (in 2006, in 2007–2011, in 2011).

The situation of the SLF introduction in Korea was estimated as the import of the seedlings of *A. alitissima*, the main host plant of SLF, and it is very relevant to the initial spread that the seedlings possibly harboring SLF egg masses were imported in large quantities at that time [13,15]. As a result of performing the ABC analysis, the initial spread of dominant groups (similar to Shanghai) spread to Korea collected in 2010 and 2011 did not consist of sequential geographic paths in Korea. In other words, it seems that their spread was not a step-by-step spread that occurs naturally by migration, but that it occurred simultaneously in two places in the north and the middle of Korea by artificial transportation, i.e., hitchhiking [57,58]. Initially, a certain group very similar to the population in the Chongming Island area in Shanghai, where CN10-SH were collected, predominantly spread throughout Korea. The area surveyed in Shanghai was an artificial reforestation area of Chongming Island, planted for landscaping, with many traces of egg mass, and high habitat density (Figure S8). In addition, the Shanghai population was believed to have moved more north in latitude, which is more genetically close to the groups of the geographically distant Shandong and Jiangsu rather than that of the geographically close Zhejiang. The spread of SLF seems to be evidence of human intervention, and in fact, *A. altissima* seedlings were brought into Korea and divided into a nursery in one place (communication with Animal Plant Quarantine Agency, Korea). The seedlings were transported to geographically distant regions in Korea, and the spread of the SLF introduced in the form of the egg mass was a cause that could occur simultaneously [13,20]. The initial spread process of this SLF exactly corresponded with the results of our ABC analysis.

Population genetics studies of this invasion propagation process may be necessary for regions where SLF has invaded, particularly in North America, because they can help identify unnatural spreads that are anthropogenic outcomes [57]. Their ecological characteristics are those that do not appear in the native area and have the characteristic of being explosively spread out in the invasion region. As in Korea, certain genetically simple groups have spread throughout the country despite having a bottleneck. This largely seems to be due to the distribution of egg masses through the transport of host plants or artifacts affected by hitchhiking [58], so identifying the movement or distribution of host plants can help to track their dispersion process in combination with the population genetics approach.

## Figures and Tables

**Table 1:** Collection data for *L. delicatula* analyzed for microsatellite data in this study.

Pop. ID	Country	Collection Site	No.	GPS-N	GPS-E	Date
KR06-CA	Korea	Cheonan, CN	16	36.44.01.6	127.15.08.1	15-Sep-2006
KR07-SL	Korea	Seoul	9	37.33.44.1	127.05.12.1	26-Sep-2007
KR08-SG	Korea	Seoul and Gyeonggi	32	37.23.52.1	126.57.26.1	20-Aug-2008
KR09-GG	Korea	Gyeonggi	44	37.38.37.1	127.14.10.1	30-Jul-2009
KR09-JE	Korea	Jeongeub, JB	9	35.28.55.1	126.53.12.1	23-Sep-2009
KR09-GB	Korea	Gyeongbuk	5	35.20.22.1	128.47.19.1	20-Jul-2009
KR09-GW	Korea	Gangwon	23	37.21.29.1	127.54.12.1	24-Aug-2009
KR10-SL	Korea	Seoul	20	37.33.42.6	126.56.39.3	28-Jun-2010
KR10-IN	Korea	Incheon, GG	40	37.47.29.9	126.16.14.4	3-Jul-2010
KR10-NY	Korea	Namyangju, GG	15	37.37.02.6	127.13.29.9	22-Aug-2010
KR10-SW	Korea	Suwon, GG	20	37.16.07.7	126.59.06.7	17-Aug-2010
KR10-IC	Korea	Icheon, GG	15	37.20.12.9	127.26.29.5	14-Jul-2010
KR10-AS	Korea	Anseong, GG	14	37.04.33.1	127.07.57.2	30-Sep-2010
KR10-CA	Korea	Cheonan, CN	22	36.44.01.6	127.15.08.1	19-Aug-2010
KR10-CY	Korea	Cheongyang, CN	20	36.26.15.8	126.46.05.0	14-Sep-2010
KR10-NS	Korea	Nonsan, CN	20	36.13.20.6	127.00.55.1	29-Sep-2010
KR10-GC	Korea	Gochang, JB	20	35.25.50.8	126.43.09.7	20-Aug-2010
KR10-GJ	Korea	Gimje, JB	20	35.48.28.7	126.59.40.7	20-Aug-2010
KR10-BA	Korea	Buan, JB	20	35.40.36.9	126.44.24.8	20-Aug-2010
KR10-KJ	Korea	Gwangju, JN	9	35.08.06.8	126.55.52.3	4-Sep-2010
KR10-AD	Korea	Andong, GB	11	36.32.31.1	128.47.49.4	31-Jul-2010
KR10-YC	Korea	Yechoen, GB	20	36.39.56.0	128.31.12.0	6-Aug-2010
KR10-SJ	Korea	Sangju, GB	17	36.22.35.3	128.08.30.7	4-Sep-2010
KR11-CW	Korea	Changwon, GN	16	37.19.41.1	127.56.47.1	5-Aug-2011
KR11-SC	Korea	Samcheok, GW	16	37.14.28.1	129.00.46.2	29-Jun-2011
CN09-BJ	China	Beijing	10	39.54.16.8	116.24.29.5	22-Jul-2009
CN10-TJ	China	Tianjin	16	39.07.15.1	117.12.54.1	5-Jul-2010
CN11-YT	China	Yantai, SD	24	37.31.40.3	121.21.27.9	15-Aug-2011
CN11-HY	China	Haiyang, SD	20	36.56.59.5	121.03.28.8	16-Aug-2011
CN11-QD	China	Qingdao, SD	24	36.19.21.6	120.23.36.9	15-Aug-2011
CN11-LY	China	Linyi, SD	20	35.43.25.9	118.32.47.1	17-Aug-2011
CN11-RZ	China	Rihzhao, SD	24	35.28.44.2	119.28.15.6	16-Aug-2011
CN11-LG	China	Lianyungang, JS	24	34.35.53.1	119.10.46.9	18-Aug-2011
CN10-SH	China	Shanghai	26	31.37.23.4	121.23.50.2	4-Sep-2010
CN10-NB	China	Ningbo, ZJ	20	29.47.42.4	121.47.37.4	5-Sep-2010
CN10-TT	China	Tiantai, ZJ	28	29.03.45.7	121.02.45.1	6-Sep-2010
CN10-LA	China	Linan, ZJ	40	30.14.01.9	119.43.29.0	7-Sep-2010
JP10-HS	Japan	Hakusan, IK	13	36.35.40.8	136.37.32.1	15-Sep-2010

**Table 2:** Summary statistics for microsatellite data from 40 local populations of *L. delicatula*. observed heterozygosity (*H*_o_); expected heterozygosity (*H*_e_); Hardy–Weinberg Equilibrium (HWE); gene diversity (*H*_S_); mean number of alleles (*N*_A_); allelic richness (*R*_S_). ns: non-significance in HWE (*p* > 0.05). † non-clonal multilocus genotype (MLG); * *p* values for heterozygote deficit or heterozygote excess (*p* < 0.001); § *F_IS_* multiple loci.

Pop. ID	MLG †	*H*_o_ (±s.d.)	*H*_e_ (±s.d.)	HWE	*H*_S_	*N*_A_	*R*_S_	*F_IS_* §
KR06-CA	16	0.443 (0.073)	0.495 (0.059)	ns	0.50	3.67	2.73	0.109
KR07-SL	9	0.389 (0.077)	0.471 (0.069)	ns	0.48	3.33	2.81	0.182
KR08-SG	32	0.388 (0.061)	0.445 (0.058)	ns	0.45	4.42	2.68	0.130
KR09-GG	44	0.445 (0.054)	0.474 (0.044)	ns	0.47	5.33	2.79	0.062
KR09-JE	9	0.398 (0.069)	0.404 (0.066)	ns	0.40	3.17	2.59	0.014
KR09-GB	5	0.400 (0.070)	0.513 (0.058)	ns	0.53	2.75	2.75	0.241
KR09-GW	23	0.322 (0.066)	0.401 (0.074)	* deficit	0.40	3.75	2.55	0.199
KR10-SL	20	0.417 (0.061)	0.436 (0.052)	ns	0.44	3.92	2.68	0.046
KR10-IN	40	0.440 (0.065)	0.468 (0.055)	ns	0.47	4.58	2.80	0.062
KR10-NY	15	0.450 (0.079)	0.434 (0.065)	ns	0.43	4.17	2.82	−0.039
KR10-SW	20	0.467 (0.073)	0.445 (0.068)	ns	0.44	4.25	2.77	−0.049
KR10-IC	15	0.467 (0.051)	0.480 (0.050)	ns	0.48	4.00	2.85	0.029
KR10-AS	14	0.500 (0.080)	0.463 (0.061)	ns	0.46	3.42	2.68	−0.083
KR10-CA	22	0.485 (0.071)	0.487 (0.046)	ns	0.49	4.08	2.81	0.004
KR10-CY	20	0.467 (0.061)	0.468 (0.046)	ns	0.47	4.00	2.80	0.002
KR10-NS	20	0.433 (0.052)	0.481 (0.042)	ns	0.48	4.58	2.88	0.101
KR10-GC	20	0.463 (0.082)	0.505 (0.062)	ns	0.51	4.67	3.01	0.087
KR10-GJ	20	0.407 (0.063)	0.437 (0.063)	ns	0.49	3.83	2.70	0.019
KR10-BA	20	0.442 (0.060)	0.453 (0.050)	ns	0.45	4.00	2.62	0.025
KR10-KJ	9	0.407 (0.063)	0.437 (0.063)	ns	0.44	3.08	2.59	0.071
KR10-AD	11	0.333 (0.075)	0.378 (0.069)	ns	0.38	2.50	2.21	0.124
KR10-YC	20	0.438 (0.053)	0.432 (0.046)	ns	0.43	3.25	2.45	−0.014
KR10-SJ	17	0.456 (0.076)	0.444 (0.048)	ns	0.44	2.42	2.20	−0.028
KR10-CW	16	0.172 (0.080)	0.171 (0.070)	ns	0.17	1.58	1.46	−0.006
KR11-SC	16	0.453 (0.066)	0.447 (0.063)	ns	0.45	3.50	2.63	−0.014
CN09-BJ	10	0.525 (0.072)	0.493 (0.062)	ns	0.49	3.58	2.88	−0.068
CN10-TJ	16	0.432 (0.085)	0.423 (0.069)	ns	0.42	3.00	2.40	−0.024
CN11-YT	24	0.542 (0.052)	0.558 (0.028)	ns	0.56	3.92	2.80	0.030
CN11-HY	20	0.463 (0.072)	0.497 (0.057)	ns	0.50	3.42	2.56	0.070
CN11-QD	24	0.448 (0.048)	0.500 (0.036)	ns	0.50	4.08	2.73	0.106
CN11-LY	20	0.504 (0.052)	0.489 (0.050)	ns	0.49	3.25	2.62	−0.033
CN11-RZ	24	0.479 (0.084)	0.457 (0.076)	ns	0.46	4.17	2.75	−0.050
CN11-LG	24	0.424 (0.051)	0.456 (0.051)	ns	0.46	3.92	2.67	0.073
CN10-SH	26	0.413 (0.041)	0.477 (0.048)	ns	0.48	4.67	2.77	0.136
CN10-NB	20	0.479 (0.065)	0.547 (0.068)	ns	0.55	5.08	3.31	0.127
CN10-TT	28	0.548 (0.050)	0.580 (0.060)	ns	0.58	4.83	3.20	0.058
CN10-LA	40	0.542 (0.065)	0.608 (0.060)	* deficit	0.61	7.25	3.76	0.110
JP10-HS	13	0.436 (0.060)	0.432 (0.044)	ns	0.43	2.25	2.12	−0.011

**Table 3:** Analysis of molecular variance (AMOVA) results for microsatellite data analysis of SLF grouped by four cases: (1) year (2) country (3) year and country (4) genetic structure (*K* = 4).

	Among Groups			Among Populations within Groups			Within Populations		
Case	Va	Percentage	*P*	Vb	Percentage	*p*	Vc	Percentage	*p*
1	0.02	0.48	0.3123	0.40	12.23	<0.0001	2.84	87.29	<0.0001
2	0.14	4.35	0.0067	0.34	10.10	<0.0001	2.88	85.54	<0.0001
3	0.20	6.01	0.0001	0.25	7.64	<0.0001	2.84	86.35	<0.0001
4	0.33	9.90	<0.0001	0.19	5.63	<0.0001	2.84	84.47	<0.0001

## Data Availability

Data are available upon request from the authors.

## Supplementary Materials

The following are available online at https://www.mdpi.com/article/10.3390/insects12060539/s1. Figure S1: Spatial autocorrelation analysis at 10 km distance class using Korean pops. *r* (blue line) means autocorrelation coefficient, U (dashed red) +95% confidence, L (dashed green) −95% confidence. Figure S2: Spatial autocorrelation analysis at 20 km distance class using Korean pops. *r* (blue line) means autocorrelation coefficient, U (dashed red) +95% confidence, L (dashed green) −95% confidence. Figure S3: Spatial autocorrelation analysis at 20 km distance class using Chinese pops. *r* (blue line) means autocorrelation coefficient, U (dashed red) +95% confidence, L (dashed green) −95% confidence. Figure S4: Spatial autocorrelation analysis at 10 km distance class using Chinese pops. *r* (blue line) means autocorrelation coefficient, U (dashed red) +95% confidence, L (dashed green) −95% confidence. Figure S5: Genetic structure of *L. delicatula* for 762 individuals collected from Korea, China and Japan, visualized by individual assignment. In order from the top to the bottom, each genetic structure is shown when *K* = 2, 3, 4 or 5, respectively. Figure S6: Genetic structure of *L. delicatula* for 762 individuals collected from Korea, China and Japan, visualized by group (pop.) assignment. In order from the top to the bottom, each genetic structure is shown when *K* = 2, 3, 4 or 5, respectively. Figure S7: Plots output by DIYABC showing the posterior probability (y-axis) of the three scenarios through the logistic regression (left) and direct estimate (right) approaches as output by DIYABC. The x-axis corresponds to the different *n_δ_* values used in the computations. Figure S8: Photographs of a collection site of CN10-SH as strongly inferred to the main source area; (A) planted density of the host plants (*A. alitissima*), (B) egg masses on a tree bark, (C) large number of SLF adults on a tree. Table S1: Pairwise *F*_ST_ divergences between 38 different geographical populations of the SLF. Pairwise G test values are above the diagonal and pairwise *F*_ST_ values are below the diagonal. Red and bold *F*_ST_ values mean those significantly different from zero at *p* < 0.001. Table S2: Results of the bottleneck test based on the two mutation models, SMM and TPM, using a nonparametric Wilcoxon signed-rank test. Table S3: Mean assignment rate of individuals into (rows) and from (columns) each population using GeneClass 2 (Piry et al. 2004). Values in bold and underlined indicate the proportions of individuals assigned to the source population. Zero values were excluded from the table. The cells were gradiented according to the size range of the numbers (see note).
