# Supplementary material for: Tracing the Origin of Korean Invasive Populations of the Spotted Lanternfly, Lycorma delicatula (Hemiptera: Fulgoridae)
Source: Insects. 2021 Jun 10;12(6):539. doi: 10.3390/insects12060539 (PMC8227202; doi:10.3390/insects12060539)
Supplement: Supplementary file 1 [file insects-12-00539-s001.zip › Supplementary material 1 - Figures S1-S8.pdf]

Tracing the origin of Korean invasive populations of the spotted lanternfly, *Lycorma delicatula* (Hemiptera: Fulgoridae)

Hyojoong Kim [1,*,†], Sohee Kim [1,2,†], Yerim Lee [1,*], Heung-Sik Lee [2], Seong-Jin Lee [2] and Jong-Ho Lee [2]

1 Animal Systematics Laboratory, Department of Biology, Kunsan National University, Gunsan, Jeonbuk 54150, Republic of Korea
2 Animal & Plant Quarantine Agency, Gimcheon, Gyeongbuk 39660, Republic of Korea
† These authors are equally contributed
* Correspondence: HK: hkim@kunsan.ac.kr; YL: yleeii@snu.ac.kr

Supplementary material 1

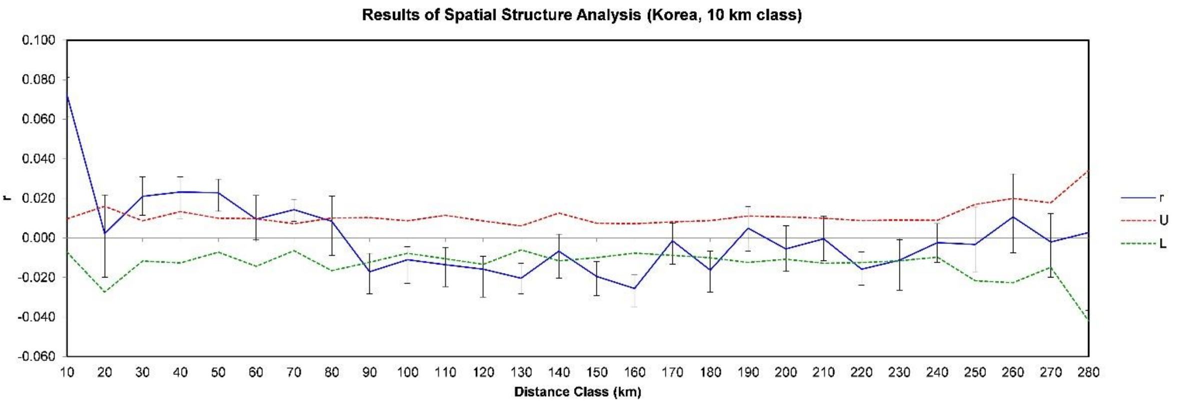

Figure S1. Spatial autocorrelation analysis at 10 km distance class using Korean pops. *r* (blue line) means autocorrelation coefficient, U (dashed red) +95% confidence, L (dashed green) -95% confidence

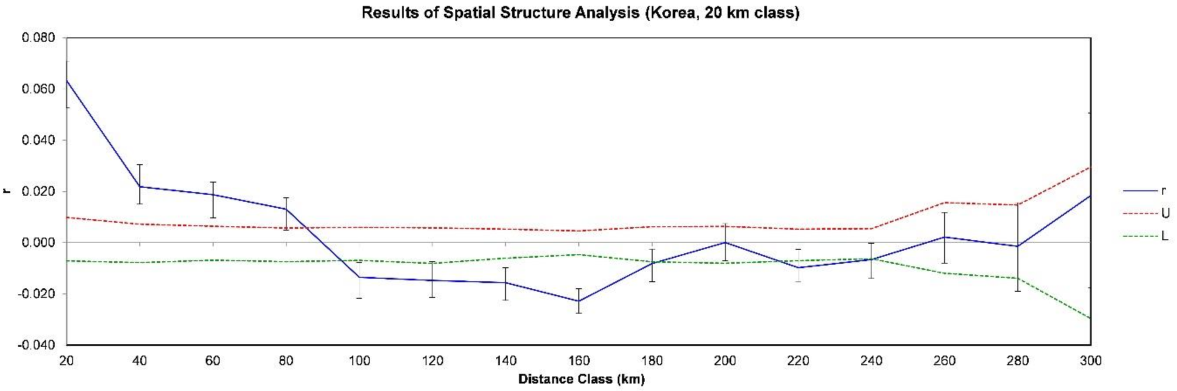

Figure S2. Spatial autocorrelation analysis at 20 km distance class using Korean pops. *r* (blue line) means autocorrelation coefficient, U (dashed red) +95% confidence, L (dashed green) -95% confidence

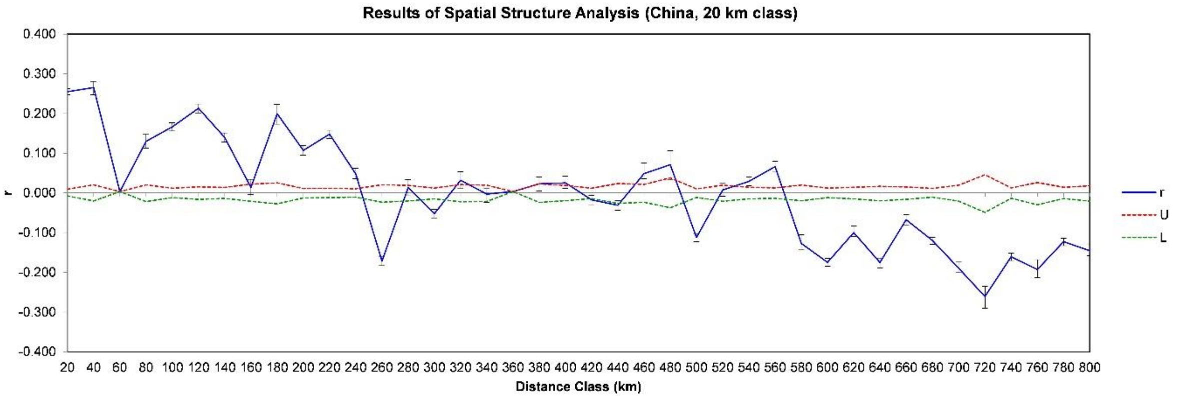

Figure S3. Spatial autocorrelation analysis at 20 km distance class using Chinese pops. *r* (blue line) means autocorrelation coefficient, U (dashed red) +95% confidence, L (dashed green) -95% confidence

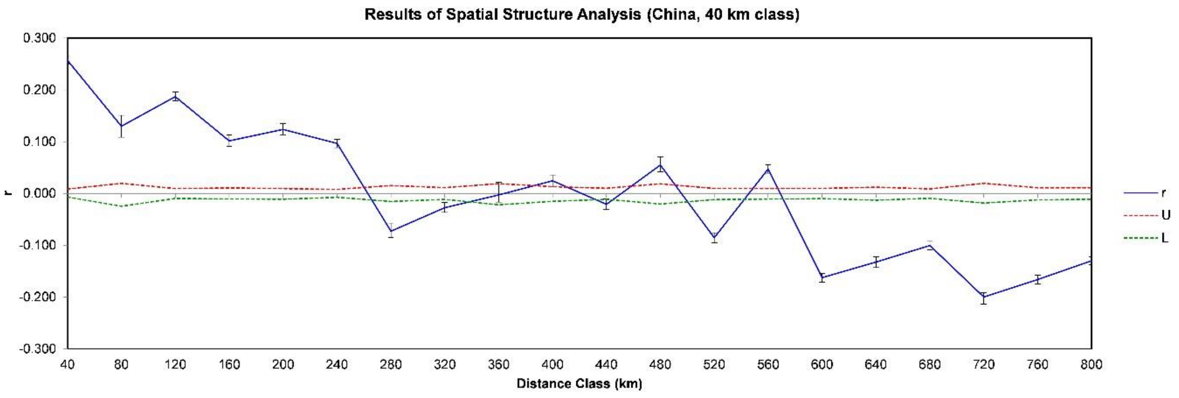

Figure S4. Spatial autocorrelation analysis at 10 km distance class using Chinese pops. *r* (blue line) means autocorrelation coefficient, U (dashed red) +95% confidence, L (dashed green) -95% confidence

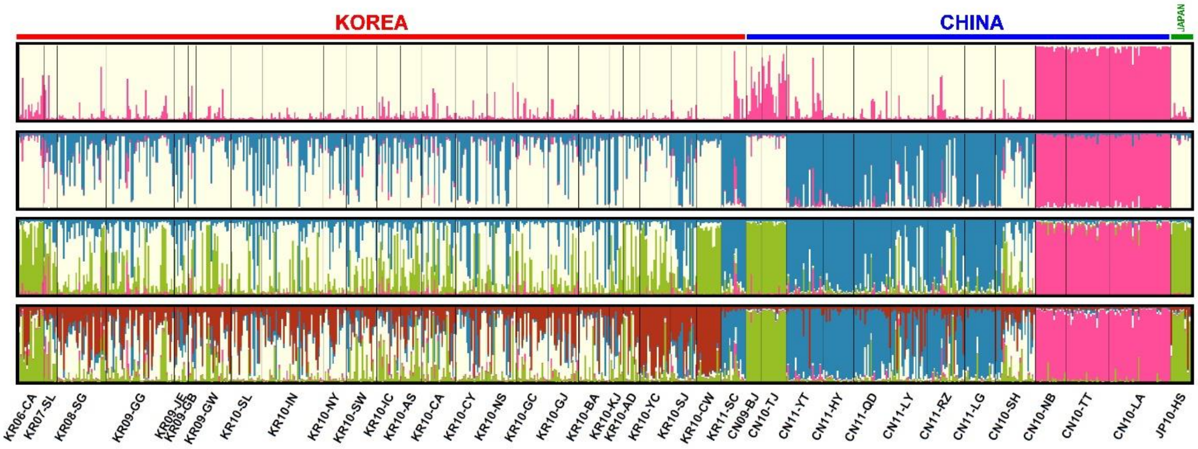

Figure S5. Genetic structure of *L. delicatula* for 762 individuals collected from Korea, China and Japan, visualized by individual assignment. In order from the top to the bottom, each genetic structure is shown when $K = 2$ (white/pink), 3 (white/pink/blue), 4 (white/pink/blue/green) or 5 (white/pink/blue/red), respectively.

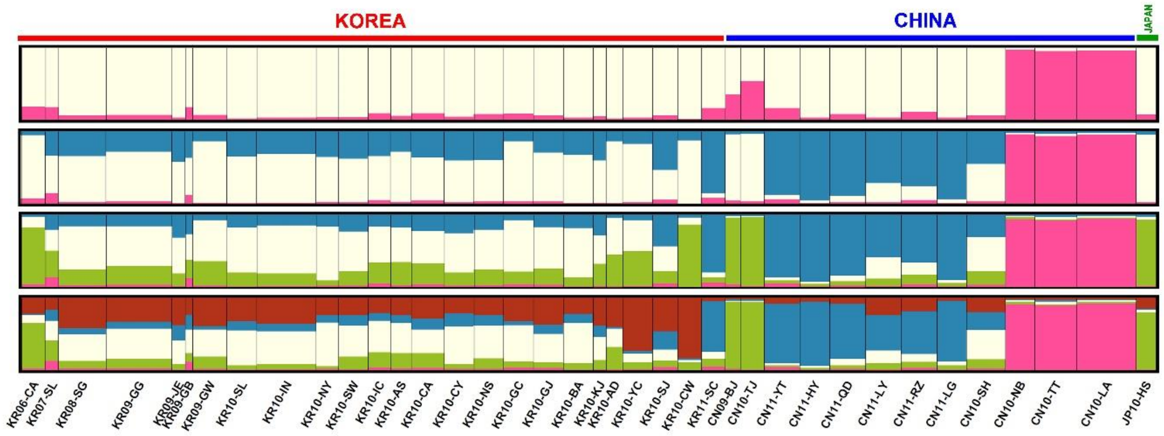

Figure S6. Genetic structure of *L. delicatula* for 762 individuals collected from Korea, China and Japan, visualized by group (pop.) assignment. In order from the top to the bottom, each genetic structure is shown when $K = 2$ (white/pink), 3 (white/pink/blue), 4 (white/pink/blue/green) or 5 (white/pink/blue/red), respectively.

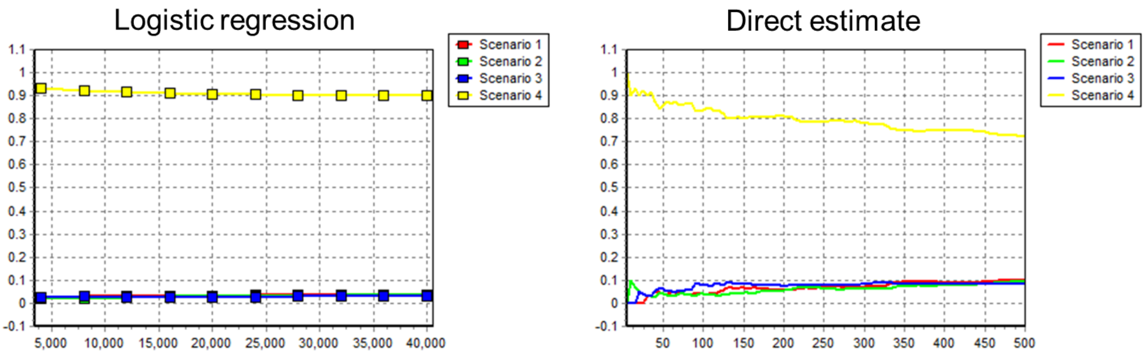

Figure S7. Plots output by DIYABC showing the posterior probability (y-axis) of the three scenarios through the logistic regression (left) and direct estimate (right) approaches as output by DIYABC. The x-axis corresponds to the different nδ values used in the computations.

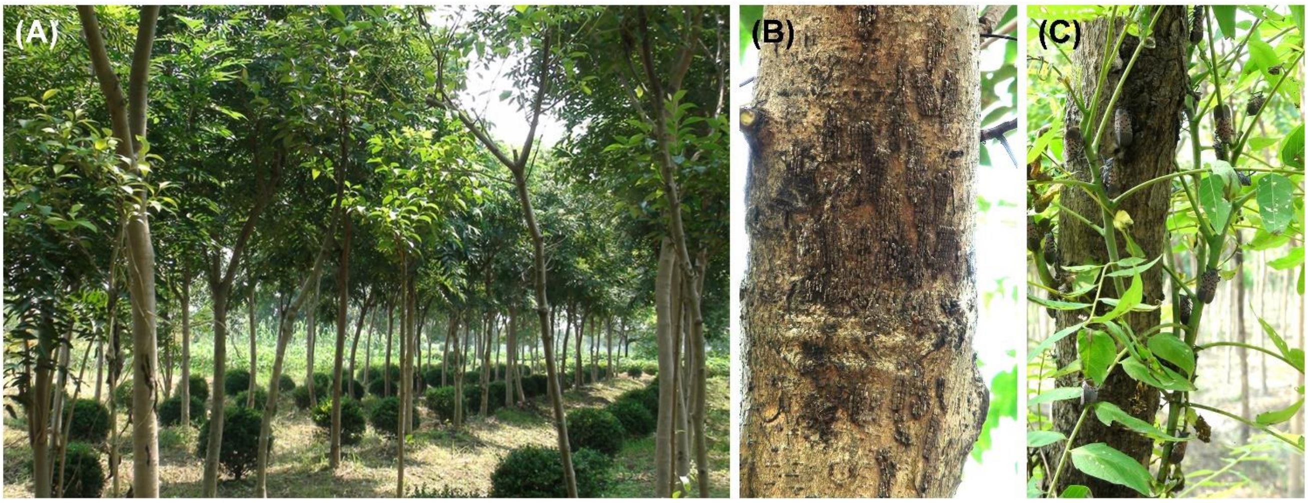

Figure S8. Photographs of a collection site of CN10-SH as strongly inferred to the main source area; (A) planted density of the host plants (*A. alitissima*), (B) egg masses on a tree bark, (C) large number of SLF adults on a tree.
