# Supplementary material for: Tracing the Origin of Korean Invasive Populations of the Spotted Lanternfly, Lycorma delicatula (Hemiptera: Fulgoridae)
Source: Insects. 2021 Jun 10;12(6):539. doi: 10.3390/insects12060539 (PMC8227202; doi:10.3390/insects12060539)
Supplement: Supplementary file 1 [file insects-12-00539-s001.zip › Supplementary material 2 - Tables S1-S3.pdf]

Tracing the origin of Korean invasive populations of the spotted lanternfly, *Lycorma delicatula* (Hemiptera: Fulgoridae)

Hyojoong Kim [1,*,†], Sohee Kim [1,2,†], Yerim Lee [1,*], Heung-Sik Lee [2], Seong-Jin Lee [2] and Jong-Ho Lee [2]

[1] Animal Systematics Laboratory, Department of Biology, Kunsan National University, Gunsan, Jeonbuk 54150, Republic of Korea
[2] Animal & Plant Quarantine Agency, Gimcheon, Gyeongbuk 39660, Republic of Korea
[†] These authors are equally contributed
[*] Correspondence: HK: hkim@kunsan.ac.kr; YL: yleeii@snu.ac.kr

Supplementary material 2

Table S1. Pairwise F_{ST} divergences between 38 different geographical populations of the SLF. Pairwise G test values are above the diagonal and pairwise F_{ST} values are below the diagonal. Red and bold F_{ST} values mean those significantly different from zero at $P < 0.001$.

	KR06-CA	KR07-SL	KR08-SG	KR09-GG	KR09-JE	KR09-GB	KR09-GW	KR10-SL	KR10-IN	KR10-NY	KR10-SW	KR10-IC	KR10-AS	KR10-CA	KR10-CY	KR10-NS	KR10-GC	KR10-GJ	KR10-BA	KR10-KJ
/ KR06-CA	-	2.568	1.567	2.036	1.978	2.339	1.882	1.539	1.900	1.779	2.160	2.576	3.084	2.833	2.083	2.058	2.236	2.298	1.628	2.233
/ KR07-SL	0.089	-	2.823	3.270	6.579	6.571	3.177	2.976	3.358	3.929	2.949	2.686	3.759	5.834	5.536	4.872	2.270	2.967	3.000	2.594
/ KR08-SG	0.138	0.081	-	21.302	10.132	2.839	13.906	29.619	16.665	6.281	4.851	4.447	4.929	6.296	5.222	13.191	8.371	7.391	24.022	18.819
/ KR09-GG	0.109	0.071	0.012	-	13.517	9.554	10.357	102.631	15.583	12.922	12.077	19.686	15.483	22.898	13.456	67.870	11.886	13.411	24.093	18.379
/ KR09-JE	0.112	**0.037**	**0.024**	**0.018**	-	7.319	18.951	12.505	46.566	Infinity	12.269	6.019	9.802	11.114	20.497	61.025	6.868	5.049	12.887	8.834
/ KR09-GB	0.097	**0.037**	0.081	**0.026**	**0.033**	-	2.316	4.724	5.228	9.671	5.060	7.419	8.151	8.017	25.234	21.661	4.401	12.023	5.018	4.237
/ KR09-GW	0.117	0.073	0.018	0.024	**0.013**	0.097	-	12.584	8.434	9.429	5.639	4.503	4.678	7.162	4.587	6.901	4.964	4.162	5.834	6.743
/ KR10-SL	0.140	0.077	**0.008**	**0.002**	**0.020**	0.050	0.019	-	22.813	13.374	33.903	14.319	7.476	10.792	12.120	247.275	9.917	11.672	94.447	32.951
/ KR10-IN	0.116	0.069	0.015	0.016	**0.005**	0.046	0.029	**0.011**	-	15.493	8.908	5.384	5.819	7.587	9.037	21.913	8.510	7.173	21.209	10.361
/ KR10-NY	0.123	0.060	0.038	0.019	**-0.004**	**0.025**	0.026	0.018	0.016	-	7.521	5.494	6.836	5.761	7.737	11.999	7.841	7.987	9.962	10.620
/ KR10-SW	0.104	0.078	0.049	0.020	**0.020**	0.047	0.042	**0.007**	0.027	0.032	-	Infinity	28.820	18.269	17.405	19.544	8.749	8.682	12.083	15.910
/ KR10-IC	0.088	0.085	0.053	**0.013**	0.040	**0.033**	0.053	**0.017**	0.044	0.044	**-0.003**	-	Infinity	Infinity	44.633	31.760	11.042	9.006	11.460	24.700
/ KR10-AS	0.075	0.062	0.048	0.016	0.025	**0.030**	0.051	0.032	0.041	0.035	**0.009**	**-0.004**	-	567.932	19.718	29.547	15.745	18.351	23.905	80.919
/ KR10-CA	0.081	0.041	0.038	0.011	0.022	**0.030**	0.034	0.023	0.032	0.042	0.014	**-0.000**	**0.000**	-	454.295	126.013	7.875	8.280	10.754	9.726
/ KR10-CY	0.107	0.043	0.046	0.018	**0.012**	**0.010**	0.052	0.020	0.027	0.031	0.014	**0.006**	**0.013**	**0.001**	-	Infinity	8.182	5.903	16.608	8.449
/ KR10-NS	0.108	0.049	**0.019**	**0.004**	**0.004**	**0.011**	0.035	**0.001**	**0.011**	**0.020**	**0.013**	**0.008**	**0.008**	**0.002**	**-0.010**	-	12.771	8.891	Infinity	19.373
/ KR10-GC	0.101	0.099	0.029	0.021	0.035	0.054	0.048	0.025	0.029	0.031	0.028	0.022	**0.016**	0.031	0.030	0.019	-	17.087	39.686	79.368
/ KR10-GJ	0.098	0.078	0.033	0.018	0.047	**0.020**	0.057	0.021	0.034	0.030	0.028	0.027	**0.013**	0.029	0.041	0.027	**0.014**	-	18.951	Infinity
/ KR10-BA	0.133	0.077	**0.010**	**0.010**	**0.019**	0.047	0.041	0.003	0.012	0.024	0.020	0.021	**0.010**	0.023	0.015	**-0.002**	**0.006**	**0.013**	-	Infinity
/ KR10-KJ	0.101	0.088	**0.013**	**0.013**	**0.028**	**0.056**	**0.036**	**0.008**	**0.024**	**0.023**	**0.015**	**0.010**	**0.003**	0.025	0.029	**0.013**	**0.003**	**-0.004**	**-0.003**	-
/ KR10-AD	0.121	0.071	0.074	0.049	0.052	0.049	**0.067**	0.040	0.056	0.074	0.038	0.055	0.047	0.046	0.048	0.028	0.101	0.061	0.059	0.058
/ KR10-YC	0.121	0.082	0.059	0.067	0.085	0.093	0.095	0.090	0.087	0.117	0.111	0.100	0.057	0.063	0.089	0.065	0.093	0.054	0.072	0.064
/ KR10-SJ	0.159	0.092	0.088	0.092	0.105	0.055	0.130	0.095	0.073	0.086	0.135	0.137	0.113	0.112	0.112	0.085	0.106	0.052	0.088	0.080
/ KR11-CW	0.261	0.283	0.183	0.221	0.289	0.408	0.219	0.240	0.191	0.289	0.289	0.307	0.301	0.260	0.284	0.244	0.272	0.266	0.248	0.272
/ KR11-SC	0.147	0.102	0.115	0.098	0.065	0.121	0.115	0.099	0.087	0.078	0.071	0.069	0.065	0.077	0.075	0.079	0.081	0.111	0.084	0.081
/ CN09-BJ	0.105	0.124	0.173	0.165	0.154	0.133	0.195	0.172	0.153	0.178	0.140	0.145	0.137	0.132	0.141	0.139	0.148	0.138	0.160	0.150
/ CN10-TJ	0.192	0.163	0.227	0.203	0.212	0.164	0.251	0.209	0.213	0.233	0.175	0.178	0.176	0.165	0.152	0.160	0.194	0.193	0.198	0.210
/ CN11-YT	0.151	0.122	0.108	0.104	0.108	0.089	0.136	0.103	0.091	0.104	0.118	0.102	0.107	0.095	0.095	0.080	0.088	0.099	0.101	0.102
/ CN11-HY	0.164	0.177	0.130	0.127	0.123	0.127	0.159	0.140	0.100	0.113	0.146	0.136	0.131	0.137	0.132	0.114	0.094	0.130	0.124	0.132
/ CN11-QD	0.162	0.113	0.077	0.067	0.065	**0.049**	0.119	0.070	0.045	0.080	0.076	0.072	0.076	0.074	0.048	0.038	0.072	0.078	0.050	0.085
/ CN11-LY	0.161	0.087	0.086	0.071	0.069	0.059	0.121	0.077	0.073	0.078	0.073	0.073	0.074	0.071	0.061	0.061	0.069	0.069	0.066	0.093
/ CN11-RZ	0.182	0.099	0.071	0.060	0.057	0.065	0.102	0.058	0.051	0.076	0.065	0.083	0.077	0.060	0.057	0.041	0.069	0.076	0.055	0.095
/ CN11-LG	0.165	0.096	0.052	0.039	0.050	0.077	0.072	0.037	0.045	0.071	0.048	0.052	0.063	0.033	0.034	0.023	0.044	0.075	0.040	0.076
/ CN10-SH	0.105	0.068	0.021	0.015	**0.003**	**0.037**	0.037	0.017	**0.006**	**0.022**	**0.019**	**0.017**	**0.010**	**0.015**	**0.008**	**-0.004**	**0.012**	0.032	**-0.000**	**0.015**
/ CN10-NB	0.201	0.230	0.257	0.249	0.263	0.206	0.293	0.279	0.239	0.254	0.265	0.232	0.218	0.224	0.224	0.220	0.198	0.226	0.225	0.221
/ CN10-TT	0.198	0.186	0.196	0.192	0.202	0.145	0.234	0.213	0.198	0.194	0.207	0.185	0.168	0.177	0.171	0.165	0.148	0.171	0.172	0.170
/ CN10-LA	0.228	0.239	0.248	0.243	0.264	0.187	0.292	0.264	0.251	0.254	0.259	0.221	0.218	0.225	0.224	0.218	0.201	0.218	0.224	0.217
/ JP10-HS	0.140	0.111	0.099	0.098	0.125	0.111	0.115	0.129	0.129	0.150	0.147	0.131	0.102	0.097	0.130	0.097	0.142	0.111	0.127	0.123

Table S1. (continued)

	KR10-AD	KR10-YC	KR10-SJ	KR11-CW	KR11-SC	CN09-BJ	CN10-TJ	CN11-YT	CN11-HY	CN11-QD	CN11-LY	CN11-RZ	CN11-LG	CN10-SH	CN10-NB	CN10-TT	CN10-LA	JP10-HS
/ KR06-CA	1.809	1.821	1.322	0.707	1.450	2.134	1.051	1.405	1.270	1.290	1.304	1.122	1.263	2.130	0.991	1.012	0.846	1.535
/ KR07-SL	3.280	2.791	2.464	0.632	2.190	1.758	1.280	1.791	1.160	1.967	2.622	2.270	2.358	3.409	0.836	1.094	0.797	1.996
/ KR08-SG	3.133	4.001	2.594	1.115	1.925	1.191	0.853	2.055	1.672	3.018	2.671	3.254	4.573	11.886	0.723	1.025	0.757	2.272
/ KR09-GG	4.817	3.468	2.462	0.879	2.290	1.270	0.983	2.157	1.726	3.481	3.267	3.948	6.150	16.676	0.755	1.054	0.779	2.290
/ KR09-JE	4.604	2.691	2.141	0.614	3.569	1.371	0.929	2.055	1.776	3.581	3.349	4.131	4.743	84.784	0.700	0.991	0.696	1.753
/ KR09-GB	4.903	2.446	4.267	0.363	1.817	1.633	1.273	2.569	1.717	4.855	4.004	3.599	3.001	6.532	0.962	1.478	1.088	2.001
/ KR09-GW	3.487	2.369	1.667	0.891	1.929	1.033	0.747	1.583	1.318	1.843	1.818	2.210	3.201	6.481	0.603	0.820	0.607	1.915
/ KR10-SL	6.030	2.517	2.369	0.790	2.278	1.199	0.947	2.166	1.529	3.327	3.012	4.089	6.540	14.430	0.647	0.925	0.698	1.681
/ KR10-IN	4.226	2.622	3.164	1.057	2.632	1.389	0.924	2.502	2.255	5.286	3.172	4.617	5.309	39.307	0.798	1.013	0.748	1.692
/ KR10-NY	3.150	1.880	2.670	0.615	2.967	1.155	0.824	2.164	1.967	2.857	2.955	3.056	3.282	11.124	0.733	1.041	0.733	1.419
/ KR10-SW	6.279	1.999	1.596	0.616	3.269	1.530	1.175	1.863	1.467	3.041	3.194	3.614	4.958	12.812	0.695	0.960	0.717	1.447
/ KR10-IC	4.297	2.246	1.570	0.564	3.352	1.470	1.152	2.192	1.591	3.234	3.164	2.755	4.566	14.370	0.829	1.104	0.879	1.657
/ KR10-AS	5.075	4.166	1.958	0.580	3.593	1.576	1.172	2.089	1.653	3.030	3.151	2.990	3.733	24.236	0.899	1.238	0.895	2.206
/ KR10-CA	5.180	3.727	1.972	0.710	2.978	1.648	1.262	2.393	1.577	3.132	3.279	3.921	7.226	16.037	0.865	1.164	0.860	2.328
/ KR10-CY	4.936	2.550	1.991	0.631	3.065	1.520	1.394	2.388	1.642	4.916	3.828	4.147	7.206	31.966	0.866	1.208	0.864	1.678
/ KR10-NS	8.753	3.625	2.700	0.774	2.914	1.555	1.312	2.894	1.947	6.301	3.875	5.921	10.841	Infinity	0.886	1.267	0.895	2.338
/ KR10-GC	2.220	2.449	2.110	0.668	2.820	1.436	1.037	2.595	2.421	3.244	3.349	3.373	5.434	20.583	1.013	1.435	0.996	1.510
/ KR10-GJ	3.829	4.385	4.539	0.688	1.999	1.564	1.046	2.278	1.675	2.952	3.370	3.050	3.070	7.624	0.858	1.208	0.896	1.997
/ KR10-BA	4.003	3.237	2.606	0.757	2.722	1.309	1.015	2.229	1.759	4.753	3.519	4.263	5.963	Infinity	0.862	1.202	0.866	1.716
/ KR10-KJ	4.025	3.676	2.873	0.669	2.848	1.420	0.939	2.213	1.641	2.686	2.430	2.385	3.040	16.932	0.880	1.220	0.903	1.786
/ KR10-AD	-	2.827	1.983	0.612	1.492	1.227	0.964	1.379	0.921	1.928	1.465	2.003	2.046	3.822	0.606	0.798	0.624	2.354
/ KR10-YC	0.081	-	2.424	0.934	1.032	1.329	0.874	1.338	1.000	1.775	1.629	1.841	1.801	2.844	0.692	0.868	0.700	3.378
/ KR10-SJ	0.112	0.093	-	0.674	1.184	0.946	0.704	2.405	1.410	1.847	1.483	1.661	1.473	2.448	0.820	1.048	0.856	1.588
/ KR11-CW	0.290	0.211	0.271	-	0.460	0.520	0.335	0.650	0.524	0.669	0.523	0.575	0.596	0.792	0.340	0.391	0.368	0.578
/ KR11-SC	0.144	0.195	0.174	0.352	-	1.117	0.863	2.341	1.697	1.917	1.924	1.709	1.940	4.093	0.828	1.201	0.824	0.955
/ CN09-BJ	0.169	0.158	0.209	0.325	0.183	-	4.719	1.298	1.031	1.362	1.390	1.315	1.136	1.588	0.750	0.909	0.682	0.995
/ CN10-TJ	0.206	0.222	0.262	0.427	0.225	0.050	-	0.938	0.649	0.968	1.001	1.046	0.971	1.117	0.650	0.934	0.675	0.751
/ CN11-YT	0.153	0.157	0.094	0.278	0.096	0.161	0.210	-	6.162	3.056	2.163	2.074	2.331	3.261	1.001	1.602	1.133	1.316
/ CN11-HY	0.214	0.200	0.151	0.323	0.128	0.195	0.278	0.039	-	3.476	2.421	1.688	1.834	3.003	0.794	1.124	0.870	0.914
/ CN11-QD	0.115	0.123	0.119	0.272	0.115	0.155	0.205	0.076	0.067	-	9.329	5.186	4.302	7.166	0.788	1.047	0.848	1.235
/ CN11-LY	0.146	0.133	0.144	0.323	0.115	0.152	0.200	0.104	0.094	0.026	-	5.894	5.005	3.914	0.676	0.977	0.775	1.149
/ CN11-RZ	0.111	0.120	0.131	0.303	0.128	0.160	0.193	0.108	0.129	0.046	0.041	-	21.320	4.782	0.619	0.920	0.668	1.157
/ CN11-LG	0.109	0.122	0.145	0.295	0.114	0.180	0.205	0.097	0.120	0.055	0.048	0.012	-	7.075	0.648	0.932	0.713	1.285
/ CN10-SH	0.061	0.081	0.093	0.240	0.058	0.136	0.183	0.071	0.077	0.034	0.060	0.050	0.034	-	0.906	1.275	0.879	1.950
/ CN10-NB	0.292	0.265	0.234	0.423	0.232	0.250	0.278	0.200	0.239	0.241	0.270	0.288	0.278	0.216	-	3.263	4.215	0.659
/ CN10-TT	0.239	0.224	0.193	0.390	0.172	0.216	0.211	0.135	0.182	0.193	0.204	0.214	0.212	0.164	0.071	-	5.117	0.913
/ CN10-LA	0.286	0.263	0.226	0.405	0.233	0.268	0.270	0.181	0.223	0.228	0.244	0.272	0.260	0.221	0.056	0.047	-	0.757
/ JP10-HS	0.096	0.069	0.136	0.302	0.207	0.201	0.250	0.160	0.215	0.168	0.179	0.178	0.163	0.114	0.275	0.215	0.248	-

Table S2. Results of the bottleneck test based on the two mutation models, SMM and TPM, using a nonparametric Wilcoxon signed-rank test

Pop. ID	Wilcowon signed-rank tests		Mode shift	MODE-Shift									
	TPM	SMM											
KR06-CA	0.2593	0.1697	normal	0.432	0.114	0.045	0.068	0.136	0.091	0.045	0.000	0.023	0.045
KR07-SL	0.1030	0.0615	shifted mode	0.282	0.308	0.077	0.051	0.026	0.103	0.000	0.128	0.000	0.026
KR08-SG	0.0320	0.0067	normal	0.566	0.113	0.057	0.019	0.038	0.038	0.075	0.038	0.019	0.038
KR09-GG	0.0031	0.0012	normal	0.609	0.125	0.031	0.047	0.031	0.031	0.047	0.031	0.047	0.000
KR09-JE	0.0415	0.0415	normal	0.351	0.216	0.081	0.081	0.000	0.000	0.135	0.054	0.054	0.027
KR09-GB	0.4251	0.2847	shifted mode	0.000	0.212	0.303	0.061	0.061	0.152	0.030	0.061	0.061	0.061
KR09-GW	0.0415	0.0061	normal	0.477	0.159	0.068	0.068	0.023	0.023	0.091	0.000	0.045	0.045
KR10-SL	0.0053	0.0017	normal	0.426	0.149	0.149	0.000	0.064	0.021	0.043	0.043	0.085	0.021
KR10-IN	0.1018	0.0212	normal	0.491	0.182	0.073	0.055	0.018	0.018	0.073	0.036	0.018	0.036
KR10-NY	0.0046	0.0017	normal	0.490	0.204	0.041	0.041	0.020	0.061	0.020	0.020	0.102	0.000
KR10-SW	0.0031	0.0006	normal	0.549	0.118	0.020	0.078	0.039	0.039	0.059	0.020	0.020	0.059
KR10-IC	0.0171	0.0040	normal	0.417	0.208	0.042	0.063	0.063	0.042	0.042	0.063	0.042	0.021
KR10-AS	0.2119	0.0881	normal	0.293	0.293	0.024	0.049	0.122	0.049	0.049	0.024	0.073	0.024
KR10-CA	0.0647	0.0067	normal	0.429	0.204	0.082	0.041	0.020	0.061	0.061	0.061	0.041	0.000
KR10-CY	0.0212	0.0212	normal	0.375	0.271	0.063	0.042	0.021	0.021	0.083	0.083	0.000	0.042
KR10-NS	0.0171	0.0031	normal	0.564	0.091	0.109	0.018	0.018	0.018	0.073	0.091	0.000	0.018
KR10-GC	0.0320	0.0031	normal	0.482	0.196	0.054	0.054	0.036	0.036	0.071	0.036	0.000	0.036
KR10-GJ	0.1506	0.1167	normal	0.391	0.174	0.065	0.087	0.087	0.022	0.109	0.000	0.022	0.043
KR10-BA	0.0053	0.0023	normal	0.479	0.104	0.104	0.063	0.021	0.021	0.104	0.042	0.042	0.021
KR10-KJ	0.0615	0.0415	shifted mode	0.250	0.306	0.028	0.083	0.056	0.083	0.083	0.028	0.056	0.028
KR10-AD	0.3501	0.2886	normal	0.207	0.207	0.103	0.069	0.069	0.069	0.138	0.034	0.000	0.103
KR10-YC	0.3667	0.2593	normal	0.308	0.205	0.103	0.051	0.026	0.026	0.128	0.077	0.051	0.026
KR10-SJ	0.9739	0.9243	shifted mode	0.172	0.034	0.207	0.069	0.103	0.207	0.034	0.103	0.000	0.069
KR10-CW	0.7813	0.7188	shifted mode	0.154	0.000	0.231	0.154	0.077	0.077	0.000	0.154	0.000	0.154
KR11-SC	0.1030	0.0337	normal	0.463	0.122	0.073	0.073	0.024	0.098	0.024	0.049	0.049	0.024
CN09-BJ	0.0874	0.0615	normal	0.286	0.286	0.048	0.119	0.048	0.024	0.119	0.048	0.000	0.024
CN10-TJ	0.5508	0.3823	normal	0.314	0.114	0.143	0.143	0.029	0.057	0.057	0.086	0.029	0.029
CN11-YT	0.7881	0.6614	normal	0.383	0.128	0.106	0.064	0.106	0.085	0.128	0.000	0.000	0.000
CN11-HY	0.7114	0.5508	normal	0.375	0.050	0.175	0.075	0.125	0.050	0.100	0.050	0.000	0.000
CN11-QD	0.2119	0.1167	normal	0.429	0.163	0.102	0.041	0.041	0.082	0.041	0.082	0.020	0.000
CN11-LY	0.8669	0.7881	normal	0.231	0.231	0.103	0.103	0.051	0.103	0.077	0.026	0.077	0.000
CN11-RZ	0.2593	0.1506	normal	0.440	0.180	0.080	0.080	0.020	0.060	0.060	0.000	0.020	0.060
CN11-LG	0.2119	0.1697	normal	0.404	0.191	0.128	0.043	0.000	0.021	0.085	0.043	0.085	0.000
CN10-SH	0.0320	0.0040	normal	0.571	0.125	0.036	0.018	0.054	0.054	0.054	0.054	0.000	0.036
CN10-NB	0.0757	0.0386	normal	0.475	0.180	0.098	0.066	0.082	0.016	0.000	0.033	0.016	0.033
CN10-TT	0.5452	0.1167	normal	0.466	0.155	0.138	0.069	0.052	0.017	0.052	0.034	0.017	0.000
CN10-LA	0.0031	0.0009	normal	0.598	0.195	0.069	0.034	0.034	0.011	0.034	0.000	0.011	0.011
JP10-HS	0.9998	0.9988	shifted mode	0.077	0.192	0.077	0.154	0.077	0.154	0.154	0.077	0.038	0.000

Table S3. Mean assignment rate of individuals into (rows) and from (columns) each population using GeneClass 2 (Piry et al. 2004). Values in bold and underlined indicate the proportions of individuals assigned to the source population. Zero values were excluded from the table. The cells were gradiented according to the size range of the numbers (see note)

	KR06-CA	KR07-SL	KR08-SG	KR09-GG	KR09-JE	KR09-GB	KR09-GW	KR10-SL	KR10-IN	KR10-NY	KR10-SW	KR10-IC	KR10-AS	KR10-CA	KR10-CY	KR10-NS	KR10-GC	KR10-GJ	KR10-BA	KR10-KJ
/ KR06-CA	<u>**0.401**</u>	0.126	0.030	0.152	0.032	0.156	0.059	0.025	0.130	0.053	0.144	0.187	0.146	0.161	0.111	0.134	0.199	0.082	0.074	0.059
/ KR07-SL	0.085	<u>**0.257**</u>	0.166	0.213	0.144	0.257	0.039	0.136	0.176	0.212	0.152	0.179	0.141	0.215	0.241	0.276	0.201	0.174	0.156	0.083
/ KR08-SG	0.120	0.214	<u>**0.421**</u>	0.456	0.243	0.284	0.250	0.351	0.385	0.330	0.324	0.315	0.266	0.328	0.318	0.442	0.468	0.298	0.327	0.234
/ KR09-GG	0.141	0.180	0.303	<u>**0.436**</u>	0.181	0.302	0.146	0.306	0.381	0.312	0.316	0.400	0.315	0.339	0.321	0.416	0.374	0.289	0.276	0.182
/ KR09-JE	0.144	0.372	0.445	0.418	<u>**0.285**</u>	0.297	0.221	0.322	0.422	0.365	0.452	0.284	0.223	0.318	0.386	0.422	0.478	0.275	0.279	0.190
/ KR09-GB	0.051	0.161	0.179	0.303	0.140	<u>**0.133**</u>	0.022	0.121	0.225	0.178	0.173	0.184	0.241	0.184	0.262	0.335	0.291	0.344	0.122	0.166
/ KR09-GW	0.193	0.309	0.426	0.401	0.299	0.283	<u>**0.382**</u>	0.353	0.385	0.377	0.342	0.328	0.239	0.432	0.322	0.398	0.534	0.253	0.280	0.267
/ KR10-SL	0.114	0.240	0.428	0.457	0.230	0.332	0.257	<u>**0.398**</u>	0.433	0.376	0.392	0.429	0.298	0.338	0.416	0.542	0.515	0.367	0.386	0.271
/ KR10-IN	0.118	0.208	0.294	0.389	0.201	0.264	0.183	0.271	<u>**0.460**</u>	0.296	0.333	0.299	0.235	0.330	0.328	0.350	0.371	0.265	0.291	0.199
/ KR10-NY	0.104	0.246	0.264	0.309	0.216	0.293	0.198	0.292	0.313	<u>**0.373**</u>	0.245	0.257	0.206	0.210	0.276	0.386	0.399	0.283	0.281	0.206
/ KR10-SW	0.110	0.204	0.255	0.355	0.182	0.248	0.153	0.279	0.341	0.251	<u>**0.384**</u>	0.392	0.255	0.251	0.277	0.368	0.418	0.265	0.244	0.186
/ KR10-IC	0.095	0.141	0.204	0.382	0.068	0.232	0.112	0.282	0.251	0.201	0.300	<u>**0.364**</u>	0.275	0.350	0.265	0.391	0.315	0.229	0.189	0.146
/ KR10-AS	0.151	0.220	0.217	0.421	0.153	0.296	0.083	0.263	0.276	0.291	0.344	0.410	<u>**0.419**</u>	0.304	0.291	0.406	0.396	0.294	0.252	0.211
/ KR10-CA	0.160	0.247	0.169	0.336	0.150	0.307	0.156	0.196	0.295	0.209	0.324	0.385	0.255	<u>**0.437**</u>	0.352	0.378	0.281	0.224	0.215	0.144
/ KR10-CY	0.074	0.202	0.209	0.330	0.195	0.320	0.111	0.226	0.328	0.310	0.305	0.340	0.254	0.302	<u>**0.431**</u>	0.451	0.364	0.259	0.287	0.139
/ KR10-NS	0.052	0.160	0.229	0.332	0.171	0.301	0.097	0.321	0.260	0.306	0.259	0.317	0.187	0.281	0.349	<u>**0.367**</u>	0.336	0.201	0.244	0.096
/ KR10-GC	0.058	0.074	0.155	0.213	0.121	0.163	0.091	0.121	0.147	0.127	0.148	0.158	0.114	0.102	0.138	0.212	<u>**0.366**</u>	0.216	0.137	0.112
/ KR10-GJ	0.106	0.183	0.217	0.398	0.147	0.348	0.118	0.243	0.340	0.247	0.309	0.355	0.294	0.270	0.303	0.371	0.512	<u>**0.420**</u>	0.247	0.276
/ KR10-BA	0.036	0.175	0.315	0.396	0.218	0.289	0.169	0.342	0.444	0.326	0.332	0.365	0.326	0.248	0.377	0.505	0.566	0.321	<u>**0.350**</u>	0.243
/ KR10-KJ	0.073	0.139	0.202	0.265	0.176	0.299	0.081	0.211	0.298	0.291	0.230	0.259	0.267	0.212	0.228	0.393	0.479	0.323	0.268	<u>**0.296**</u>
/ KR10-AD	0.140	0.293	0.349	0.483	0.270	0.438	0.217	0.494	0.453	0.364	0.504	0.423	0.340	0.420	0.448	0.612	0.432	0.399	0.448	0.282
/ KR10-YC	0.090	0.263	0.239	0.405	0.199	0.291	0.128	0.244	0.278	0.188	0.197	0.285	0.336	0.321	0.293	0.408	0.383	0.304	0.212	0.231
/ KR10-SJ	0.077	0.254	0.209	0.336	0.141	0.547	0.048	0.247	0.373	0.200	0.227	0.251	0.230	0.216	0.273	0.418	0.399	0.519	0.186	0.266
/ KR11-CW	0.491	0.550	0.824	0.712	0.487	0.334	0.629	0.654	0.796	0.551	0.476	0.506	0.518	0.627	0.508	0.752	0.688	0.331	0.662	0.627
/ KR11-SC	0.016	0.096	0.126	0.102	0.125	0.118	0.054	0.111	0.085	0.152	0.184	0.194	0.093	0.086	0.136	0.152	0.157	0.084	0.090	0.072
/ CN09-BJ	0.016	0.029	0.009	0.015	0.001	0.033	0.005	0.002	0.003	0.007	0.010	0.018	0.001	0.009	0.015	0.046	0.042	0.005	0.010	0.002
/ CN10-TJ	0.070	0.074	0.013	0.063	0.008	0.130	0.017	0.022	0.036	0.022	0.086	0.107	0.047	0.066	0.087	0.143	0.096	0.049	0.047	0.029
/ CN11-YT	0.007	0.010	0.035	0.050	0.018	0.086	0.009	0.049	0.055	0.046	0.026	0.075	0.030	0.064	0.048	0.121	0.057	0.028	0.023	0.010
/ CN11-HY	0.005	0.030	0.055	0.066	0.034	0.114	0.009	0.059	0.075	0.112	0.058	0.057	0.031	0.028	0.059	0.153	0.102	0.042	0.026	0.018
/ CN11-QD	0.029	0.080	0.129	0.132	0.116	0.184	0.052	0.148	0.172	0.150	0.179	0.196	0.148	0.102	0.197	0.243	0.172	0.146	0.140	0.079
/ CN11-LY	0.008	0.157	0.146	0.107	0.123	0.138	0.019	0.122	0.134	0.186	0.200	0.124	0.096	0.037	0.141	0.214	0.213	0.137	0.081	0.073
/ CN11-RZ	0.015	0.138	0.192	0.188	0.122	0.205	0.096	0.171	0.141	0.157	0.148	0.111	0.078	0.140	0.090	0.273	0.233	0.131	0.093	0.128
/ CN11-LG	0.025	0.109	0.247	0.257	0.154	0.233	0.133	0.204	0.246	0.214	0.251	0.184	0.121	0.207	0.180	0.325	0.341	0.153	0.139	0.133
/ CN10-SH	0.085	0.151	0.250	0.328	0.176	0.230	0.118	0.249	0.333	0.280	0.276	0.332	0.248	0.232	0.248	0.393	0.371	0.177	0.260	0.167
/ CN10-NB	0.001	0.000	0.000	0.000	0.000	0.002	0.000	0.000	0.000	0.000	0.000	0.000	0.000	0.000	0.000	0.000	0.001	0.000	0.000	0.000
/ CN10-TT	0.000	0.000	0.000	0.000	0.000	0.004	0.000	0.000	0.000	0.001	0.000	0.000	0.000	0.000	0.001	0.002	0.004	0.000	0.000	0.000
/ CN10-LA	0.000	0.000	0.000	0.000	0.000	0.001	0.000	0.000	0.000	0.000	0.000	0.000	0.001	0.000	0.000	0.000	0.000	0.000	0.000	0.000
/ JP10-HS	0.144	0.125	0.174	0.264	0.117	0.243	0.117	0.131	0.149	0.119	0.133	0.186	0.201	0.173	0.142	0.252	0.187	0.122	0.154	0.127

Table S3. (continued)

	KR10-AD	KR10-YC	KR10-SJ	KR11-CW	KR11-SC	CN09-BJ	CN10-TJ	CN11-YT	CN11-HY	CN11-QD	CN11-LY	CN11-RZ	CN11-LG	CN10-SH	CN10-NB	CN10-TT	CN10-LA	JP10-HS
/ KR06-CA	0.001	0.027	0.003	0.000	0.014	0.136	0.005	0.012	0.019	0.020	0.004	0.007	0.018	0.174	0.015	0.002	0.039	0.002
/ KR07-SL	0.027	0.077	0.048	0.000	0.039	0.169	0.011	0.048	0.015	0.116	0.108	0.042	0.078	0.260	0.002	0.003	0.024	0.004
/ KR08-SG	0.074	0.147	0.015	0.000	0.098	0.132	0.015	0.173	0.107	0.261	0.179	0.216	0.262	0.377	0.005	0.014	0.043	0.028
/ KR09-GG	0.056	0.110	0.045	0.000	0.056	0.103	0.017	0.161	0.060	0.194	0.112	0.149	0.205	0.389	0.004	0.007	0.031	0.022
/ KR09-JE	0.080	0.145	0.031	0.000	0.148	0.137	0.011	0.241	0.107	0.300	0.198	0.252	0.290	0.466	0.007	0.007	0.027	0.021
/ KR09-GB	0.002	0.133	0.105	0.000	0.075	0.042	0.003	0.113	0.063	0.199	0.189	0.189	0.156	0.186	0.004	0.004	0.078	0.000
/ KR09-GW	0.068	0.092	0.017	0.000	0.086	0.169	0.029	0.144	0.058	0.181	0.138	0.207	0.231	0.370	0.004	0.013	0.027	0.018
/ KR10-SL	0.096	0.105	0.038	0.000	0.131	0.126	0.012	0.202	0.084	0.258	0.194	0.196	0.264	0.396	0.002	0.009	0.035	0.006
/ KR10-IN	0.081	0.081	0.041	0.006	0.087	0.109	0.011	0.149	0.096	0.247	0.123	0.138	0.166	0.360	0.004	0.004	0.029	0.006
/ KR10-NY	0.074	0.088	0.125	0.000	0.107	0.100	0.012	0.183	0.112	0.164	0.151	0.163	0.179	0.268	0.004	0.010	0.030	0.009
/ KR10-SW	0.063	0.062	0.033	0.000	0.087	0.104	0.010	0.101	0.043	0.178	0.110	0.139	0.193	0.364	0.002	0.006	0.033	0.002
/ KR10-IC	0.033	0.050	0.000	0.000	0.082	0.108	0.017	0.082	0.015	0.102	0.083	0.130	0.176	0.307	0.007	0.005	0.043	0.000
/ KR10-AS	0.016	0.110	0.005	0.000	0.088	0.083	0.008	0.193	0.053	0.169	0.139	0.089	0.158	0.367	0.004	0.017	0.079	0.014
/ KR10-CA	0.037	0.081	0.007	0.000	0.031	0.117	0.013	0.080	0.022	0.112	0.067	0.056	0.135	0.304	0.003	0.002	0.024	0.002
/ KR10-CY	0.020	0.027	0.003	0.000	0.113	0.074	0.005	0.173	0.066	0.203	0.139	0.093	0.168	0.352	0.005	0.005	0.032	0.000
/ KR10-NS	0.050	0.066	0.002	0.000	0.035	0.062	0.006	0.117	0.037	0.183	0.080	0.102	0.149	0.317	0.007	0.008	0.028	0.005
/ KR10-GC	0.011	0.043	0.011	0.000	0.014	0.045	0.002	0.029	0.009	0.043	0.023	0.050	0.100	0.166	0.004	0.006	0.018	0.001
/ KR10-GJ	0.063	0.137	0.073	0.000	0.031	0.089	0.012	0.111	0.030	0.156	0.130	0.164	0.180	0.277	0.002	0.005	0.023	0.050
/ KR10-BA	0.034	0.050	0.010	0.000	0.045	0.069	0.003	0.150	0.102	0.244	0.100	0.172	0.224	0.437	0.005	0.013	0.053	0.000
/ KR10-KJ	0.020	0.078	0.090	0.000	0.063	0.080	0.006	0.167	0.056	0.184	0.091	0.110	0.159	0.289	0.004	0.009	0.072	0.002
/ KR10-AD	**0.358**	0.235	0.113	0.000	0.067	0.166	0.045	0.139	0.027	0.198	0.058	0.163	0.167	0.423	0.002	0.009	0.017	0.021
/ KR10-YC	0.062	**0.410**	0.045	0.000	0.051	0.125	0.006	0.079	0.034	0.154	0.070	0.180	0.161	0.291	0.002	0.003	0.018	0.011
/ KR10-SJ	0.025	0.167	**0.461**	0.000	0.040	0.049	0.002	0.218	0.115	0.249	0.102	0.220	0.132	0.335	0.006	0.017	0.142	0.001
/ KR11-CW	0.255	0.642	0.041	**0.470**	0.270	0.643	0.076	0.451	0.302	0.583	0.320	0.440	0.411	0.717	0.008	0.004	0.046	0.122
/ KR11-SC	0.002	0.001	0.000	0.000	**0.424**	0.043	0.002	0.205	0.097	0.179	0.122	0.116	0.104	0.226	0.002	0.026	0.050	0.000
/ CN09-BJ	0.000	0.000	0.000	0.000	0.001	**0.361**	0.137	0.002	0.002	0.004	0.003	0.005	0.005	0.047	0.010	0.001	0.001	0.000
/ CN10-TJ	0.004	0.012	0.000	0.000	0.005	0.476	**0.442**	0.009	0.001	0.016	0.005	0.006	0.010	0.082	0.010	0.006	0.012	0.000
/ CN11-YT	0.000	0.006	0.001	0.000	0.027	0.005	0.000	**0.439**	0.128	0.135	0.034	0.049	0.043	0.112	0.004	0.012	0.062	0.000
/ CN11-HY	0.000	0.001	0.000	0.000	0.045	0.010	0.000	0.344	**0.382**	0.242	0.169	0.075	0.106	0.129	0.001	0.012	0.044	0.000
/ CN11-QD	0.012	0.024	0.022	0.000	0.066	0.036	0.001	0.233	0.185	**0.398**	0.259	0.150	0.157	0.254	0.003	0.003	0.041	0.011
/ CN11-LY	0.000	0.004	0.001	0.000	0.117	0.020	0.001	0.224	0.179	0.409	**0.432**	0.232	0.221	0.212	0.001	0.003	0.028	0.000
/ CN11-RZ	0.019	0.055	0.037	0.000	0.057	0.039	0.003	0.151	0.078	0.187	0.145	**0.425**	0.226	0.149	0.001	0.007	0.010	0.000
/ CN11-LG	0.019	0.045	0.022	0.000	0.088	0.048	0.004	0.215	0.143	0.234	0.180	0.392	**0.432**	0.281	0.001	0.006	0.014	0.001
/ CN10-SH	0.027	0.059	0.007	0.000	0.095	0.119	0.011	0.152	0.123	0.210	0.103	0.139	0.201	**0.363**	0.011	0.009	0.057	0.018
/ CN10-NB	0.000	0.000	0.000	0.000	0.000	0.000	0.000	0.000	0.000	0.000	0.000	0.000	0.000	0.000	**0.369**	0.024	0.180	0.000
/ CN10-TT	0.000	0.000	0.000	0.000	0.000	0.000	0.000	0.000	0.000	0.000	0.000	0.000	0.000	0.000	0.071	**0.446**	0.345	0.000
/ CN10-LA	0.000	0.000	0.000	0.000	0.000	0.000	0.000	0.000	0.000	0.000	0.000	0.000	0.000	0.000	0.029	0.042	**0.371**	0.000
/ JP10-HS	0.091	0.147	0.073	0.000	0.029	0.123	0.028	0.060	0.018	0.117	0.061	0.061	0.087	0.242	0.003	0.003	0.027	**0.390**

Note.

gradient	0.000 0.00	0.090 ≥ 0.001	0.100 ≥ 0.1	0.200 ≥ 0.2	0.300 ≥ 0.3	0.400 ≥ 0.4	0.500 ≥ 0.5	0.600 ≥ 0.6	0.700 ≥ 0.7	0.800 ≥ 0.8
/range		< 0.1	< 0.2	< 0.3	< 0.4	< 0.5	< 0.6	< 0.7	< 0.8	
